# Structural Flexibility of Hydrated RHO Nanosized Zeolite Synthesized via Green Synthesis Approach at Subfreezing Conditions

**DOI:** 10.1002/smtd.202501376

**Published:** 2025-09-04

**Authors:** Sajjad Ghojavand, Giorgia Confalonieri, Stoyan P. Gramatikov, Edwin B. Clatworthy, Aymeric Magisson, Diógenes Honorato Piva, Francesco Dalena, Riccardo Fantini, Rossella Arletti, PetkoSt. Petkov, Georgi N. Vayssilov, Svetlana Mintova

**Affiliations:** ^1^ Université de Caen Normandie ENSICAEN CNRS, LCS, Laboratoire Catalyse et Spectrochimie Caen 14000 France; ^2^ Department of Earth Sciences Sapienza Università di Roma Rome 00185 Italy; ^3^ Faculty of Chemistry and Pharmacy Sofia University “St. Kliment Ohridski” Sofia 1126 Bulgaria; ^4^ Chemical and Geological Sciences Department University of Modena and Reggio Emilia Via G. Campi 103 Modena 41125 Italy

**Keywords:** flexibility, gas separation, nanozeolite, subfreezing conditions

## Abstract

Understanding the structural flexibility of zeolites under cryogenic conditions is essential for optimizing gas separation and storage performance. This study investigates nanosized RHO zeolite synthesized via green synthesis (without organic structural directing agent) upon hydration and cooling to low temperatures (<273 K) using in situ XRPD, in situ FTIR spectroscopy, and DFT simulations. Template‐free synthesis is performed at low temperature (363 K), avoiding calcination or postsynthetic activation, yielding highly crystalline nanosized zeolite with minimal energy consumption and no toxic by‐products. Upon hydration at 300 K, nanosized RHO zeolite adopts a two‐phase expanded‐contracted structure due to distinct water‐cation interactions. Upon cooling to 248 K, the hydrated zeolite transitions into a single expanded phase, remaining stable after reheating to 300 K, forming a metastable state. In situ FTIR analysis indicates freezing‐induced water molecule rearrangement leads to persistent hydrogen‐bonding networks, preventing structural reversion. This metastable state exhibits CO_2_ adsorption capacities comparable to conventionally activated RHO zeolite (623 K), achieved through significantly lower energy input. This performance underscores the viability of mild, green chemistry‐aligned activation approaches eliminating energy‐intensive high‐temperature treatments. This novel approach contributes to sustainable separation processes and provides a blueprint for future innovation in porous materials guided by green chemistry principles.

## Graphical abstract

This study reveals that hydrated nanosized RHO zeolite, synthesized via a green, template‐free route, forms a metastable expanded phase upon subfreezing treatment. This phase remains stable at ambient temperature and exhibits CO_2_ adsorption comparable to high‐temperature activated samples, offering a low‐energy, sustainable alternative for gas separation aligned with green chemistry principles.

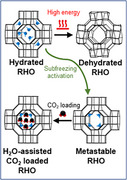

## 1 Introduction

Zeolites are microporous materials possessing framework type structures consisting of cages and channels, capable of hosting extra‐framework cations and exhibiting Lewis and Brønsted acid sites.^[^ ^1^, ^2^, ^3^ ^]^ These properties give zeolites the ability to act in key roles in industrial applications such as catalysis, gas purification, ion exchange, and potentially various other processes pertinent to addressing present environmental and economic challenges.^[^ ^3^, ^4^, ^5^ ^]^ Different approaches have been applied to control the selectivity of zeolite adsorbents and simultaneously control the adsorption and release of guest molecules.^[^ ^6^ ^]^ Capturing CO_2_, CH_4_ and NO_X_ has been of particular interest over recent decades, because of the rapid rise in the concentration of greenhouse gases in the atmosphere.^[^ ^7^, ^8^, ^9^ ^]^ While porous materials such as metal organic frameworks (MOFs), carbons, and zeolites are capable of removing pollutants from the atmosphere, the selective separation of CO_2_ from point sources, such as natural gas or fossil fuel combustion exhaust streams, is of critical importance as atmospheric CO_2_ is one of the main actor in current anthropogenic global warming.^[^ ^10^, ^11^ ^]^ The mechanical, chemical, and hydrothermal stability of zeolites, their affordable cost‐benefit ratio, and flexible frameworks makes them prime candidates for use as CO_2_ adsorbents.^[^ ^1^, ^12^, ^13^ ^]^ Flexibility in zeolites was defined as a reversible framework deformation (expansion/contraction), a dynamic of the zeolite structure, due to the movement of extra‐framework cations.^[^ ^1^ ^]^ A foremost example of zeolite flexibility can be found in RHO zeolite.^[^ ^14^, ^15^, ^16^, ^17^, ^18^, ^19^ ^]^ The RHO framework structure can undergo a distortion of the unit cell resulting in a change in symmetry from centric (*Im‐*3*m*) to acentric (*I‐*43*m*), and deformation of the double eight‐membered ring (D8R) composite building units from round to elliptical.^[^ ^20^ ^]^ This flexibility behavior is strongly linked to the physico‐chemical properties of the RHO zeolite, i.e., Si/Al ratio and cation content.^[^ ^15^ ^]^ This phenomenon has been the subject of both theoretical and experimental investigations, demonstrating a lattice volume decrease of up to 21%.^[^ ^15^, ^21^, ^22^, ^23^ ^]^ Changes to the size of the lattice have also been observed as a consequence of the adsorption of CO_2_.^[^ ^14^, ^19^, ^24^, ^25^, ^26^, ^27^, ^28^ ^]^ These changes in unit cell volume of RHO zeolite in response to CO_2_ adsorption were recently directly visualized by our group using in situ TEM imaging.^[^ ^16^ ^]^

However, the majority of studies on the flexibility of zeolite RHO are focused at room temperature or higher (i.e., 303–1073 K), whereas the effect of low temperatures (<273 K) on the flexibility of zeolite RHO is significantly under‐explored. Work by Parise et al. measured the structure of dehydrated ND_4_‐RHO and D‐RHO, and RHO loaded with deuterated methanol (CD_3_OD) at 11 K.^[^ ^29^ ^]^ Both the ND_4_‐RHO and D‐RHO exhibited distorted unit cells, however, the structure of CD_3_OD‐RHO was much less distorted. In comparison, Fischer et al. observed a less distorted unit cell for dehydrated D‐RHO at 13 K; the sample had been subjected to deep‐bed calcination resulting in a high framework Si/Al ratio.^[^ ^30^ ^]^ EPR spectroscopy by Michalik et al. on the formation of cationic silver clusters by γ‐irradiated Ag^+^‐exchanged RHO zeolite revealed more rapid formation of clusters on hydrated RHO zeolite than on dehydrated RHO zeolite at low temperatures (160 K).^[^ ^31^ ^]^ However, the cationic silver clusters were less stable at higher temperatures on the hydrated RHO zeolite. The more rapid cluster formation and lower stability were attributed to the less distorted zeolite structure at low temperatures due to the presence of water. Adsorption analyses have shown that access to the microporosity of dehydrated cation‐containing RHO zeolite (Si/Al 2–4) at cryogenic temperatures (77 K) by N_2_ and H_2_, is severely restricted.^[^ ^15^, ^32^, ^33^, ^34^ ^]^ This is due to the severe distortion of the D8Rs following the removal of water, as the extra‐framework cations seek better coordination with the framework oxygens while also occupying sites at the pore apertures.

During the adsorption of molecules onto a surface, capillary condensation can occur when vapor condensation is below the saturation vapor pressure of the pure liquid fraction. This is linked to an increase in van der Waals interactions between vapor‐phase molecules inside a confined space, such as the micropores of a zeolite.^[^ ^35^ ^]^ In other words, when molecules in the gaseous state adsorb on the zeolite surface within the micropores, at a certain loading the adsorbed gas will be present in liquid form inside the pores due to the confinement effect.^[^ ^35^ ^]^ The question we would like to answer is: “What will happen to water molecules below their melting point (273 K) in the micropores of RHO zeolite?”. Interestingly, it has been shown that free ice can exist 20 K above the melting point of water, exploiting OH stretching excitation.^[^ ^36^ ^]^ Thus, further exploration of zeolite's flexibility in the presence of water at subfreezing conditions can reveal the full potential of zeolite for gas separation and gas storage applications.^[^ ^14^, ^15^, ^16^, ^19^, ^25^, ^37^ ^]^

In this work, we explore the structural flexibility of nanosized RHO zeolite at subfreezing conditions (<273 K) conditions and the impact on its adsorption behavior. Using in situ X‐ray powder diffraction (XRPD), FTIR spectroscopy, and DFT simulations, we systematically investigate the effect of water adsorption, freezing, and re‐heating on the framework flexibility and cation dynamics in the RHO nanozeolite. Our study aims to determine whether freezing‐induced structural changes lead to a persistent metastable state, which could serve as an alternative to high‐temperature activation methods for optimizing the zeolite performance in CO_2_ capture and separation processes. The synthesis and activation of zeolites for gas separation applications must increasingly align with green chemistry principles to address environmental sustainability challenges. The green synthesis of nanosized RHO zeolite without organic structure‐directing agents (OSDA) represents a significant green chemistry advance, eliminating the need for expensive and environmentally harmful organic templates while achieving high crystalline yields. Green chemistry benefits of OSDA‐free synthesis include reduced waste generation, elimination of toxic organic compounds, and simplified processing routes that minimize environmental impact. Furthermore, nanosized zeolites offer inherent green chemistry advantages through enhanced mass transfer properties and reduced diffusion limitations, leading to improved gas separation performance compared to conventional micron‐sized materials.^[^ ^9^, ^38^ ^]^ The exploration of mild activation alternatives, such as subfreezing conditions, directly addresses green chemistry principles by developing energy‐efficient processes that operate under environmentally benign conditions while maintaining functional performance.

## 2 Experimental and Modeling Section

### 2.1 Materials and Synthesis

All chemicals were used as received without further purification, unless stated otherwise. The materials employed were LUDOX AS‐40 (SiO_2_, 40 wt. % suspension, Aldrich), sodium aluminate (NaAlO_2_, 50−56% Al_2_O_3_, 40−45% Na_2_O, Sigma–Aldrich), sodium hydroxide (NaOH, reagent grade, 98%, Sigma–Aldrich), and sodium silicate (reagent grade, 10.6% Na_2_O, 26.5% SiO_2_, Sigma–Aldrich). An aqueous solution of cesium hydroxide (CsOH, 50 wt.% Cs) was prepared by dissolving cesium hydroxide monohydrate (Alfa Aesar, 20% H_2_O, 98%) in deionized water. All water used was MilliQ from a Merck Millipore Direct‐8 ZR0Q008WW unit.

Sodium aluminate (0.518 g) was dispersed in dd water (2.11 g) under rapid stirring in a sealed 60 mL polypropylene container. Sodium hydroxide (1.795 g) and aqueous cesium hydroxide (0.393 g) were then added sequentially. After stirring the mixture for 1 h at room temperature, the solution was heated statically at 333 K for 30 min in a preheated oven. The mixture was subsequently cooled to room temperature and stirred rapidly for an additional 30 min.^[^ ^15^, ^16^ ^]^ Colloidal silica (4.77 g) was added dropwise to the solution with vigorous stirring, followed by an ageing process under continuous stirring for 48 h. The molar composition of oxides in the initial alkali aluminosilicate suspension was 0.2 Cs_2_O: 8.1 Na_2_O: 10.0 SiO_2_: 0.8 Al_2_O_3_: 90.0 H_2_O. Additional colloidal silica (3.81 g) was then introduced, followed by a dropwise addition of a mixture of sodium silicate (1.844 g) and sodium hydroxide (0.275 g), again under rapid stirring. The final suspension was stirred for another 24 h before being subjected to static hydrothermal treatment at 363 K for 2 h. The final oxide composition of the colloidal suspension was 0.2 Cs_2_O: 10.1 Na_2_O: 20.6 SiO_2_: 0.8 Al_2_O_3_: 150.2 H_2_O. The final crystalline material was obtained through a simple, environmentally friendly work‐up process: it was separated and washed with hot (90 °C) deionized water by centrifugation until the supernatant reached a neutral pH (7–8), then dried at 333 K overnight. This low‐temperature, template‐free synthesis route avoids the need for calcination or postsynthetic activation, yielding a highly crystalline nanosized zeolite product with minimal energy consumption and no generation of toxic by‐products, an approach fully aligned with green chemistry principles.

### 2.2 Characterization

Scanning Electron Microscopy (SEM) images were taken using a JEOL JSM‐IT800 Schottky field emission scanning electron microscope in high‐vacuum mode (pressure <10^−4^ Pa) at a low accelerating voltage of 0.8 keV and a current of 10nA. ImageJ software was used to analyze the particle size distribution according to the SEM images.

Inductively coupled plasma mass spectrometry (ICP‐MS) measurements were conducted with an Agilent Technologies 7900 ICP‐MS system. 50 mg of sample was subjected to a digestive media (0.5 mL aqua regia at 1:3 HNO_3_:HCl V/V, and 3 mL HF 40%), then heated to 110 °C for 1 h before being neutralized with 2 g of boric acid and made up to 100 mL.

Gravimetric water adsorption analysis was performed at 300 K using a Surface Measurements Systems Dynamic Vapor Sorption (DVS) unit. The zeolite was degassed at 623 K under high vacuum (10^−5^ torr) for 5 h. The adsorption isotherm for CO_2_ was recorded at temperatures of 300 K using a Micromeritics 3Flex Surface Characterization unit. The samples underwent an outgassing process under vacuum conditions at 623 K for a minimum of 7 h prior to measurement.

High‐resolution X‐ray powder diffraction patterns of RHO nanosized zeolite were collected during two distinct experiments at the beamline ID22 (ESRF, France)^[^ ^39^ ^]^ using the wavelengths at 29 keV [λ = 0.427715(4)Å]^[^ ^40^ ^]^ and 35 keV [λ = 0.354537(3)Å]^[^ ^41^ ^]^. All XRPD patterns were converted to the wavelengths at 35 keV [λ = 0.354537(3)Å]. Samples were loaded into 0.7 mm quartz capillary with quartz wool filled on both sides and connected to a vacuum pump. Samples were preactivated in a conventional oven at 623 K under vacuum overnight. The XRPD patterns were collected under 5 mL min^−1^ of Ar flow at 500 K for 5 h. To prevent preferred orientations, a rocking of the capillary [−15–15]° was applied during the data acquisition on transmission geometry. Data were collected up to *d* = 0.6 Å with an Eiger2 XCdTe 2M‐W detector preceded by 13 Si (111) analyzer crystals, at temperatures between 248 and 500 K; prior all measurements an equilibrium time of 30 min was applied. The oversaturation of nanosized RHO zeolite with water was performed using a water droplet placed on aluminum foil using a 0.5 mm quartz capillary and was vacuumed into the packed nanosized RHO zeolite.

TOPAS 6 was used to perform Le Bail, Pawley, and Rietveld refinement on XRPD patterns.^[^ ^42^ ^]^ Background, cell parameters, and peak profile were first refined under the Pawley refinement and then transferred to the Rietveld refinement. The refined structural parameters include the fractional coordinates (x, y, z) and isotropic displacement factors for all atoms, and the site occupancy factors (SOF).

In situ FTIR measurements of nanosized RHO zeolite was performed on a self‐supported pellet (≈14.5 mg with a diameter of 16 mm). The FTIR spectra were recorded with a Thermo Scientific Nicolet iS50 FTIR spectrometer equipped with an MCT detector, at a spectral resolution of 4 cm^−1^. The infrared cell used for the H_2_O and CO_2_ adsorption isotherm experiments was equipped with a glassware jacket for liquid N_2_ to keep the temperature inside the chamber at 100 K. A heating element in order to dehydrate the nanosized RHO zeolite at 623 K prior to the measurements. The sample cell kept under high vacuum (up to 10^−6^ kPa). Nanosized RHO zeolite was activated by heating with a ramp rate of 3 K min^−1^ followed by heating at 623 K for 4 h under high vacuum. The molar absorption coefficients used in this work to obtain the physisorption and chemisorption isotherms were 16 and 40 cm µmol^−1^, respectively.^[^ ^3^ ^]^

### 2.3 Density Functional Theory (DFT) Simulations

Quantum chemistry calculations and Ab initio Born−Oppenheimer molecular dynamic simulations were performed using the CP2K/Quickstep package.^[^ ^43^, ^44^ ^]^ Density functional theory was applied within the generalized gradient approximation (GGA), using Perdew–Burke–Ernzerhof (PBE) functional.^[^ ^45^ ^]^ Basis set DZVP‐MOLOPT‐SR‐GTH, which is optimized for calculating molecular properties in gas and condensed phase, was applied for all atoms in the studied systems.^[^ ^46^ ^]^ For reducing computational cost Gaussian and Plane‐Wave (GPW) method was used.^[^ ^44^, ^47^, ^48^ ^]^ This method uses an atom‐centered Gaussian‐type basis to describe the wave functions and an auxiliary plane wave basis to describe the electron density.^[^ ^44^ ^]^ Only the valence electrons are explicitly treated. Their interaction with the remaining ions is described using the pseudopotentials of Goedecker−Teter−Hutter (GTH).^[^ ^49^, ^50^ ^]^ The dispersion interactions are accounted for by the empirical dispersion correction of the D3 type.^[^ ^51^ ^]^

All Born–Oppenheimer molecular dynamics simulations were initially performed in the NVT ensemble, followed by the NPT_I ensemble, with a time step of 1 fs. Four different RHO unit cell models were constructed to investigate the effect of water content in the zeolite cavities:

1) A dry model without water molecules.
2) A hydrated model containing 37 water molecules.
3) A hydrated model containing 40 water molecules.
4) A hydrated model containing 46 water molecules.

For RHO structure we used the model, employed earlier for simulation of the adsorption of CO_2_ at different temperature.^[^ ^16^ ^]^ This model contains five Cs^+^ cations per unit cell, as the zeolites samples used in the experiment in the present work. In all simulations, reported here, the initial unit cell parameter was set at a = 14.80 Å and the water molecules, where available, were positioned within the two *lta* cages of the unit cell.

## 3 Results and Discussion

**Figure 1:**
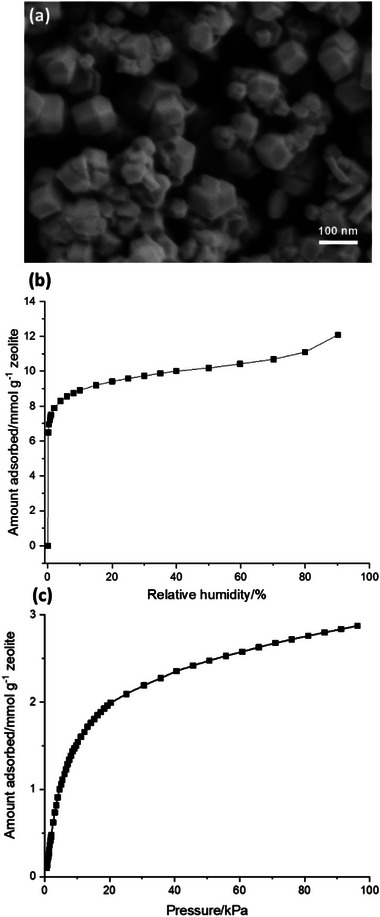
a) SEM image, b) H_2_O adsorption, and c) CO_2_ adsorption isotherms at 298 K of nanosized RHO.

The nanosized RHO zeolites with an average discrete crystal size of ≈75 nm, synthesized via a green synthetic approach with high crystalline yield (above 65%), are presented in **Figures** **1**a and S1 (Supporting Information).^[^ ^15^ ^]^ The chemical composition of nanosized RHO zeolite stored at ambient conditions based on ICP‐MS is Na_11.6_Cs_5.0_Si_31.4_Al_16.6_O_96_.37H_2_O. The nanosized RHO zeolite samples do not require calcination prior to use, aligning with the principles of green synthesis by eliminating energy consumption and avoiding CO_2_ emissions.

The gravimetric water adsorption isotherm and volumetric CO_2_ adsorption isotherm of nanosized RHO at 298 K are shown in Figure 1b,c. The H_2_O adsorption isotherm exhibits a Type I behavior, with a maximum water uptake of 12.09 mmol g^−1^ at 90% relative humidity, corresponding to 46 water molecules per unit cell (Figure 1b). Due to the strong affinity of water molecules for the zeolite surface, attributed to the high aluminum content in the framework (Si/Al = 1.9), over 75% of the total water uptake occurs below 20% relative humidity (Figure 1b).

### 3.1 Ab Initio Molecular Dynamic Predictions of RHO Zeolite Structural Flexibility at Subfreezing Conditions

First, the initial geometries of the systems were optimized (Figure S2a, Supporting Information), followed by ab initio molecular dynamics simulations. The four models were simulated at four temperatures (i.e., 250, 300, 350, and 500 K) resulting in 16 analyzed trajectories. The ellipticity parameter (∆), defined as half of the difference between the longest and shortest O…O distances across the D8R, and the unit cell parameter (a) were calculated from the final 20 ps of each simulation. To determine the space group of each model, a transition threshold (based on the literature) for the ellipticity parameter was set at ∆ = 1.35 Å. Frames with Δ < 1.35 Å were classified within the centrosymmetric *Im‐*3*m* space group, while frames with Δ > 1.35 Å were assigned to the *I‐*43*m* space group.^[^ ^15^, ^16^, ^28^ ^]^ Using this classification, the percentage of the time in which the structure corresponds to each space groups was quantified throughout the molecular dynamics (MD) simulations, as reported in the last column of **Table** **1**. Note, however, that the unit cell size varies during the simulation and the system may change its symmetry group.

**Table 1:** The thermostat temperature of the MD simulations, the average value of the ellipticity parameter Δ, the average value of the cell parameter a, the amount of the water molecules per unit cell and the estimated percentage of the predominant space group.

Model	Temperature/K	ellipticity parameter Δ/Å	a/Å	H_2_O amount (per unit cell)	Space group
1	250 K	Δ = 1.72	14.83	no water	98% *I‐*43*m*
1	300 K	Δ = 1.64	14.91	no water	94% *I‐*43*m*
1	350 K	Δ = 1.65	14.88	no water	98% *I‐*43*m*
1	500 K	Δ = 1.40	15.03	no water	64% *I‐*43*m*
2	250 K	Δ = 1.71	14.88	37	96% *I‐*43*m*
2	300 K	Δ = 1.66	14.90	37	93% *I‐*43*m*
2	350 K	Δ = 1.66	14.91	37	95% *I‐*43*m*
2	500 K	Δ = 1.47	14.99	37	68% *I‐*43*m*
3	250 K	Δ = 1.38	14.99	40	45% *I‐43m*
3	300 K	Δ = 1.42	15.03	40	59% *I‐*43*m*
3	350 K	Δ = 1.25	15.08	40	67% *Im‐*3*m*
3	500 K	Δ = 1.03	15.15	40	85% *Im‐*3*m*
4	250 K	Δ = 0.86	15.14	46	99% *Im‐*3*m*
4	300 K	Δ = 1.08	15.14	46	86% *Im‐*3*m*
4	350 K	Δ = 0.71	15.22	46	95% *Im‐*3*m*
4	500 K	Δ = 0.59	15.26	46	99% *Im‐*3*m*

The calculations indicate that in the absence of water at 250–350 K, RHO zeolite exhibits a relatively small unit cell size of ≈14.8–14.9 Å, with the D8Rs adopting an elliptical shape characterized by an ellipticity parameter (Δ) of ≈1.60–1.75 Å (see Table 1). This structural configuration corresponds to the *I‐*43*m* space group. At 500 K the average unit cell parameter of the water‐free system is somewhat larger, 15.03 Å and ellipticity parameter decreases to Δ = 1.40.

**Figure 2:**
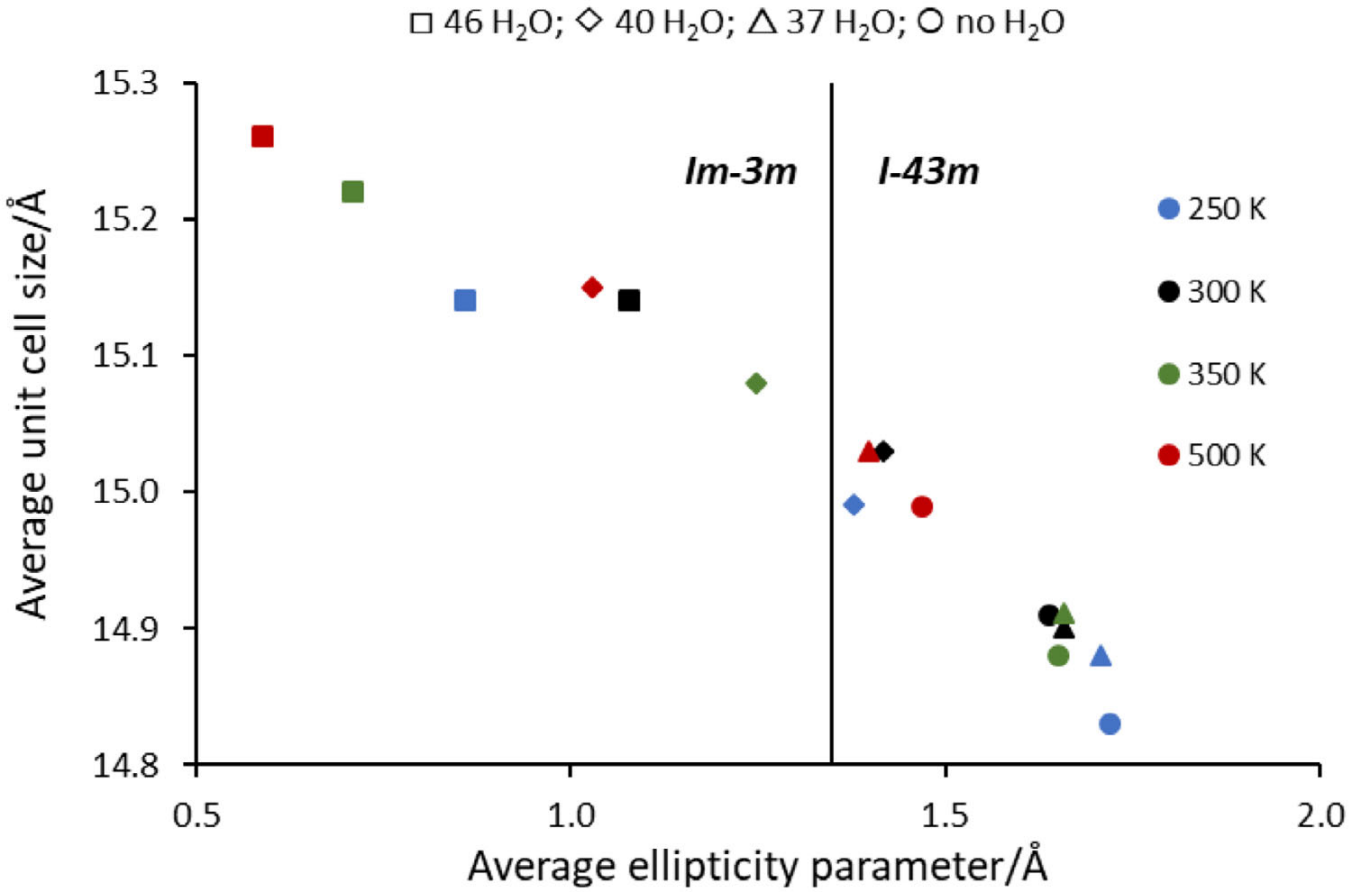
Plot of the average unit cell size (Å) versus the average parameter of ellipticity (in Å) for each simulated temperature. There are four points corresponding to the models with 0, 37, 40, and 46 water molecules. The vertical line shows the border value of the ellipticity parameter, 1.35 Å, on the base of which the symmetry group of the structure is determined.

The computational results for the hydrated model with 37 water molecules are very similar to those of the system in the absence of water at 250–350 K. The differences in the unit cell size and the ellipticity parameter are at most 0.05 and 0.02 Å, respectively (Table 1). In contrast, when the number of water molecules increases to 40 or 46 per unit cell, the zeolite predominantly adopts the *Im‐*3*m* symmetry group, accompanied by an increase in unit cell size and a decrease in the ellipticity parameter (Table 1). The extent of these changes depends on both the water content and temperature. Examining the effect of temperature, a decrease from 500 to 250 K results in a contraction of the unit cell by 0.20 Å for the water‐free model. For the hydrated systems, a temperature decrease from 350 to 250 K leads to a reduction of the cell parameter by 0.03, 0.07, and 0.08 Å in the models containing 37, 40, and 46 water molecules, respectively. Concurrently, the ellipticity parameter increases by 0.05, 0.13, and 0.15 Å (Table 1 and **Figure** **2**). These trends are consistent across all systems, regardless of water content, though the magnitude of the changes varies.

The introduction of water at 350 K leads to an expansion of the unit cell relative to the dry system, increasing by 0.20 and 0.34 Å for 40 and 46 water molecules, respectively. At 250 K, a similar trend is observed, with an increase of 0.16 and 0.31 Å. The trend of the increasing lattice parameter, decreasing ellipticity, and the transition from the *I‐*43*m* to *Im‐*3*m* space group with increasing water content is consistent with earlier computational analysis by Balestra et al. of Na‐RHO.^[^ ^52^ ^]^ Consistently, the ellipticity parameter decreases by 0.40 and 0.94 Å at 350 K and by 0.34 and 0.86 Å at 250 K for 40 and 46 water molecules, respectively (Table 1 and Figure 2). As mentioned above, the calculated structural features of the model with 37 water molecules are of the same magnitude as those of the water‐free system at all simulated temperatures. The average unit cell size of the structure containing 37 water molecules at 300 K is 14.90 Å. This value is close to the experimentally determined unit cell parameter of the second smaller RHO phase in the hydrated system at 300 K (14.84 Å), described below. The other phase, experimentally determined at the same temperature, has a cell size of 15.09 Å, which corresponds to an intermediate value between the calculated cell parameters of the models with 40 and 46 water molecules. Thus, one may suggest that the two phases observed experimentally correspond to phases with different amounts of adsorbed water molecules (vide infra—the phase with a smaller unit cell containing fewer water molecules and the phase with a larger unit cell containing more).

The difference in structural properties between the systems with 37 and 40 water molecules can be explained by variations in the interactions between Cs^+^ cations and the oxygen atoms of the water molecules. The strength of these interactions is related to the average distance between Cs^+^ and oxygen (from the water molecules) and can be described by the radial distribution function (RDF) at a given temperature. In the model with 40 water molecules, the first peak of the RDF has almost the same value at 250 and 300 K, whereas in the system with 37 water molecules, the first RDF peak at 300 K is significantly smaller than at 250 K (Figure S2b, Supporting Information). This indicates that in the system with lower water content at 300 K, water molecules show weaker Cs^+^ interactions. Of note, the position of the first peak of the RDF in the 40 water molecule system (≈3.24 and ≈3.44 Å at 250 and 300 K, respectively) is slightly larger than the experimental value determined by Lapshin and Golubeva for the bond length between the shared position of Cs^+^/Na^+^ cations and water (2.96(1) Å, 41 water molecules, 296 K), the Na,Cs‐RHO zeolite possessed a Si/Al of 3.71 and *Im‐*3*m* space group.^[^ ^53^ ^]^ Another structural feature distinguishing the hydrated systems from the water‐free model is the position of the Cs^+^ cations during molecular dynamics (MD) simulations. In the absence of water, the Cs^+^ cations remain near the center of the D8Rs, regardless of the simulated temperature. In contrast, in the hydrated systems, some Cs^+^ cations fluctuate from the center of the D8Rs toward the single eight‐membered ring (8MR) windows. Moreover, in certain MD trajectories, some Cs^+^ cations escape from the D8Rs, become solvated by water molecules, and migrate toward the interior of the *lta* cages. This is evident from the increasing distance between Cs^+^ cations and the center of the D8Rs (Figure S3, Supporting Information). These movements in the hydrated systems are consistent with the trend toward the *Im‐*3*m* space group due to the reduced coordination of the Cs^+^ cations to the zeolite framework oxygens at the 8MR or inside the *lta* cages.^[^ ^53^, ^54^ ^]^

As previously reported in studies on RHO zeolite, a correlation exists between unit cell expansion and ellipticity reduction.^[^ ^16^, ^17^, ^29^ ^]^ This relationship is further reinforced by the present simulations, which confirm that the observed structural modifications from 250 to 500 K are driven by variations in water content.

### 3.2 Structural Flexibility of Nanosized RHO Zeolite Studied by In Situ Synchrotron Radiation Powder Diffraction

**Scheme 1:**
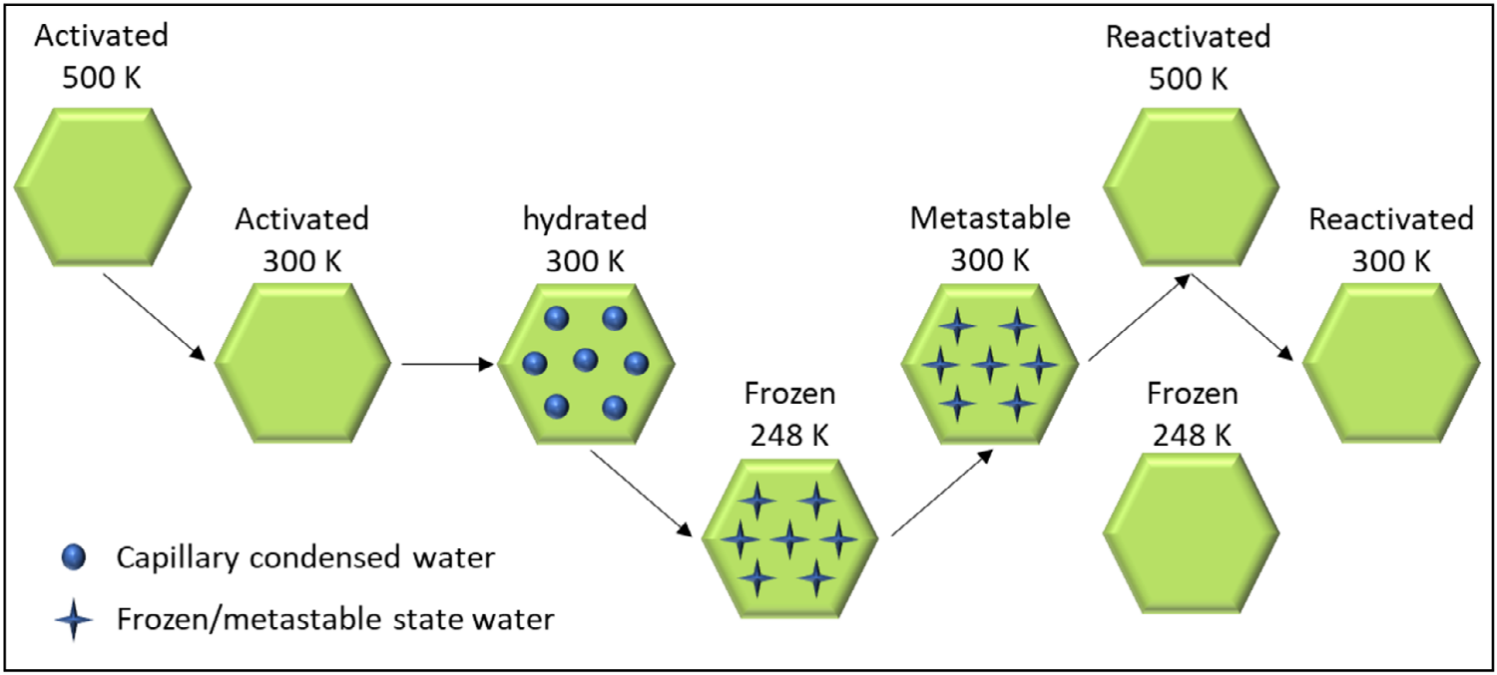
Schematic representation of the experimental procedure for in situ XRPD measurements on nanosized RHO zeolite.

**Scheme** **1** illustrates the experimental procedure used to investigate water adsorption on nanosized RHO zeolite at subfreezing conditions by in situ XRPD. The measurements performed on the nanosized RHO zeolite involved the following steps: activation at 500 K, cooling to 300 K, hydration at 300 K, and then further cooling to 248 K. Subsequently, the nanosized RHO zeolite was reheated to 300 K, reactivated at 500 K, and cooled again to 300 K. For comparison, the XRPD pattern of the activated nanosized RHO zeolite at 248 K was also recorded to evaluate the frozen hydrated sample against its non‐hydrated counterpart (Scheme 1).

**Figure 3:**
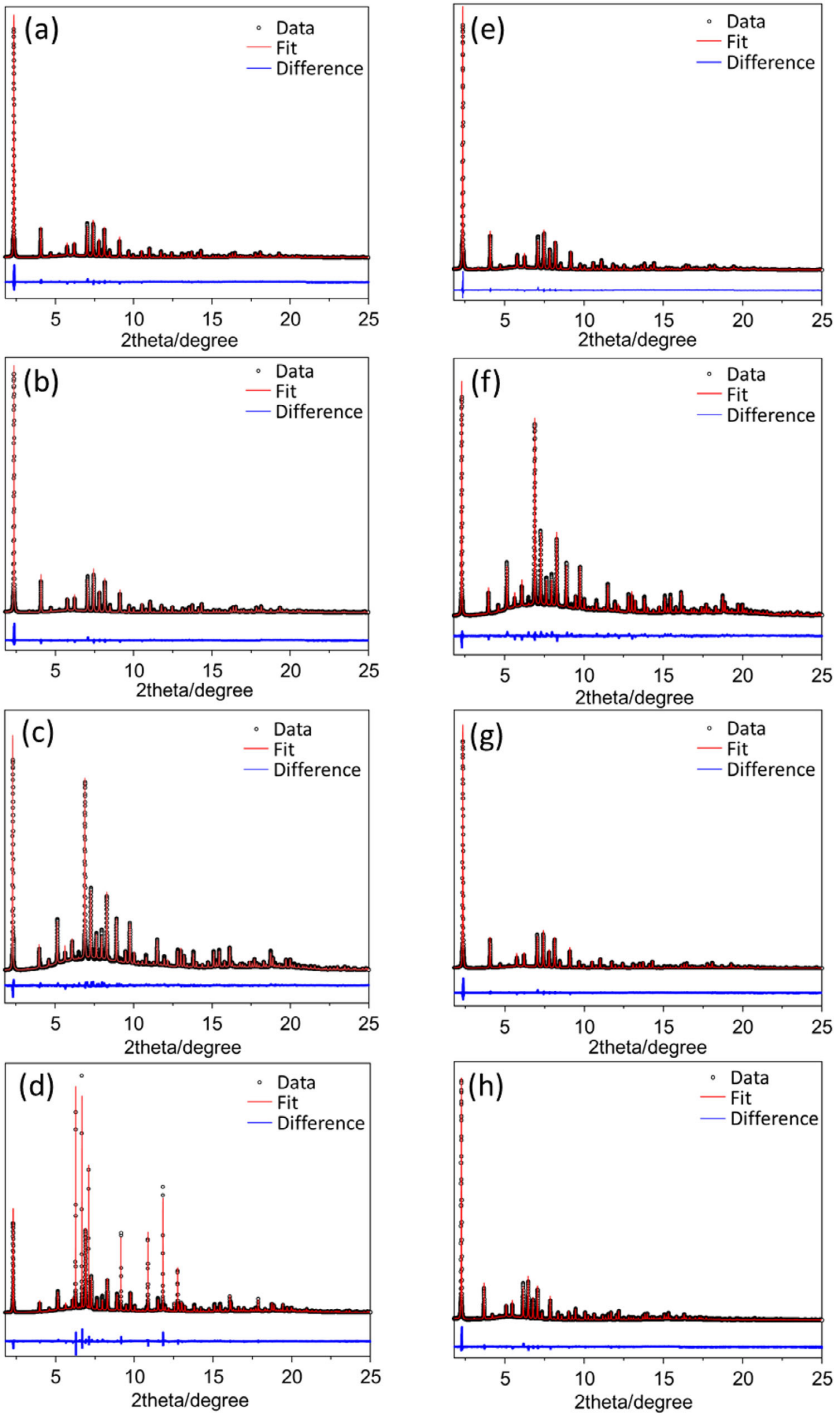
XRPD patterns and the fit deriving from Rietveld refinements of nanosized RHO zeolite a) activated at 500 K, b) activated at 300 K, c) hydrated at 300 K, d) frozen at 248 K while hydrated, e) frozen at 248 K without water, f) metastable state at 300 K, g) reactivated at 500 K, and h) reactivated at 300 K.

**Figure** **3** presents the XRPD patterns and their corresponding Rietveld refinements of nanosized RHO zeolite at the different stages depicted in Scheme 1. The lattice parameter changes and the D8R elliptical distortion of the nanosized RHO zeolite are summarized in **Table** **2**.

**Table 2:** Lattice parameter and D8R elliptical distortion of nanosized RHO zeolite after activation at 500 K, activation at 300 K, hydration at 300 K, freezing at 248 K while hydrated, freezing at 248 K without water, metastable state at 300 K, reactivation at 500 K, and reactivation at 300 K based on Rietveld refinement.

State	a/Å	Ellipticity(𝚫)/Å
Activated at 500 K	14.7834(5)	1.69
Activated at 300 K	14.7327(4)	1.68
Hydrated at 300 K	15.0931(5) 14.8390(5)^a)^	1.13 –
Frozen at 248 K while hydrated	15.09244(4)	1.08
Frozen at 248 K without water	14.6655(8)	1.72
Metastable state at 300 K	15.0936(4)	1.07
Reactivated at 500 K	14.7837(5)	1.69
Reactivated at 300 K	14.7422(4)	1.70

^a)^ Fitted by Le Bail refinement of the second phase.

**Figure 4:**
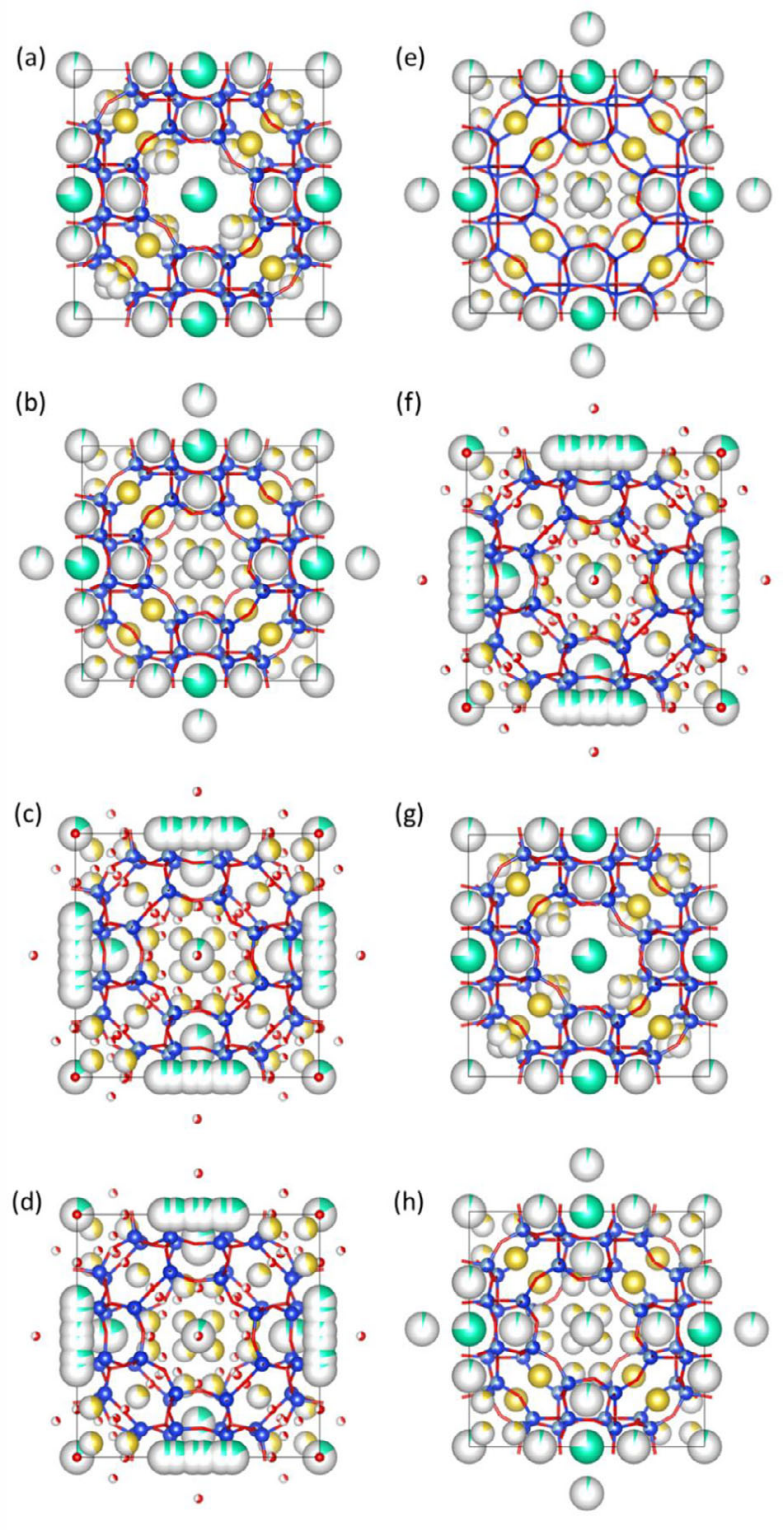
Framework structure of nanosized RHO zeolite a) activated at 500 K, b) activated at 300 K, c) hydrated at 300 K, d) frozen at 248 K while hydrated, e) frozen at 248 K without water, f) metastable state at 300 K, g) reactivated at 500 K, and h) reactivated at 300 K obtained from Rietveld refinement. Color code: blue for Si or Al, red for oxygen atom of water, yellow for Na^+^ cations, and green for Cs^+^ cations (oxygen atoms of the zeolite framework are omitted for clarity).

The XRPD patterns and Rietveld refinement of the activated nanosized RHO zeolite at 500 K are presented in Figure 3a, Table 2, and Table S1 (Supporting Information). The refined lattice parameter *a* is 14.7834(5) Å (*I‐*43*m* space group), with an ellipticity of the D8Rs of 1.69 Å, consistent with our previous studies but slightly higher than the value reported by Baur et al. (≈1.50 Å).^[^ ^14^, ^15^, ^55^ ^]^ This difference is attributed to the higher Cs^+^ content in this work and the lower Si/Al ratio (1.9 vs 3.1, respectively).^[^ ^14^, ^15^, ^55^ ^]^ **Figure** **4**a illustrates the framework of nanosized RHO activated at 500 K, derived from Rietveld refinement. Two distinct crystallographic sites for Na^+^ cations in the dehydrated state at 500 K were identified (Table S1, Supporting Information and Figure 4a). The first site accommodates 8 Na^+^ per unit cell, positioned at the center of the single six‐rings (S6Rs) in the *lta* cage. The second site contains the remaining 3.6 Na^+^ cations, located just below the S6Rs, within the *lta* cage. This distribution is consistent with our previous findings on nanosized RHO.^[^ ^14^, ^15^ ^]^ Similarly, Cs^+^ cations in activated nanosized RHO at 500 K occupy two distinct sites (Table S1, Supporting Information and Figure 4a). The first site, with low occupancy (3% of 12 equivalent sites = 0.48 Cs^+^), is located at the center of the 8MR window of the *lta* cage. The second, more dominant site (77% of 6 equivalent sites = 4.52 Cs^+^), is positioned at the center of the D8Rs. In our previous study on nanosized RHO zeolite, only a single Cs^+^ site was reported, located in the middle of the D8Rs.^[^ ^15^ ^]^ However, instead of using anisotropic atomic displacement factors to describe the Cs^+^ displacement, as in our prior work, we opted for a simplified approach by assigning distinct crystallographic locations. This distinction accounts for Cs^+^ movement both inside and outside the D8Rs, as evidenced by the two identified sites (Table S1, Supporting Information and Figure 4a).

The XRPD pattern and Rietveld refinement of activated nanosized RHO zeolite after cooling to 300 K are shown in Figure 3b and Table 2 and Table S2 (Supporting Information). The refined lattice parameter *a* decreased slightly to 14.7327(4) Å (*I‐*43*m* space group) due to thermal contraction, while the ellipticity of the D8Rs remained nearly constant at 1.68 Å. At 300 K, two distinct crystallographic locations were still observed for both Na^+^ and Cs^+^ cations (Table S2, Supporting Information). The Na^+^ cations positioned at the center of S6Rs in the *lta* cage remained largely unchanged. However, the Na^+^ cations originally located just below the S6Rs migrated closer to the center of the *lta* cage upon cooling (Table S2, Supporting Information and Figure 3b). Similarly, the Cs⁺ cationic positions remained nearly identical after cooling to 300 K (Table S2, Supporting Information and Figure 3b). However, a slight increase in Cs^+^ occupancy at the center of the D8Rs was observed, rising from 77% to 80% (Table S2, Supporting Information).

Figure 3c and Table 2 and Table S3 (Supporting Information) present the XRPD pattern and Rietveld refinement of hydrated nanosized RHO zeolite at 300 K. Analysis of the XRPD patterns revealed two distinct phases: a dominant expanded phase with a lattice parameter *a* = 15.0931(5) Å (*I‐*43*m* space group), for which Rietveld refinement was possible, and a minor contracted phase with *a* = 14.8390(5) Å, identified through Le Bail refinement of the remaining diffraction peaks. The peaks of the two phases are well separated, allowing us to structurally refine the main one (see Figure S5, Supporting Information). The coexistence of two hydration‐induced phases has also been observed in nanosized RHO zeolite with a low Si/Al ratio (1.5).^[^ ^28^ ^]^ Figure 4c displays the expanded nanosized RHO framework after Rietveld refinement, where it appears evident the decrease of D8Rs ellipticity (to 1.13 Å). Upon hydration, three distinct Cs^+^ crystallographic sites were identified (Table S3, Supporting Information and Figure 4c), all closely spaced in and around the D8Rs, indicative of Cs^+^ oscillation through the center of the D8Rs. The Na^+^ cations maintained two crystallographic sites, similar to the dehydrated state, however, a significant redistribution occurred, with more Na^+^ cations migrating from the S6Rs to the *lta* cage (1.8 vs 8.0 Na^+^). This is due to the water molecules effecting the Na^+^ cation coordination sphere, which would otherwise be satisfied by the zeolite framework oxygens (see Table S3, Supporting Information). Hydration resulted in the incorporation of 36.9 H_2_O molecules, corresponding to 40% relative humidity (RH) at 300 K (the ambient condition during the experiment). These water molecules were distributed across five distinct crystallographic sites (W1–W5, Table S3, Supporting Information): 7 molecules coordinated with Cs^+^ cations (W2), 2 molecules positioned at the center of the *lta* cages (W4), and the remaining water molecules coordinated with Na^+^ cations inside the *lta* cages (W1, W3, and W5). This distribution is consistent with our previous study on hydrated RHO.^[^ ^14^, ^15^, ^16^ ^]^ However, as previously mentioned (vide supra), instead of employing anisotropic atomic displacement factors, we describe cation motion by assigning closely spaced crystallographic sites, such as the three Cs^+^ locations, which mirror the displacement patterns obtained using anisotropic modeling in our earlier works.^[^ ^14^, ^15^, ^16^ ^]^

XRPD patterns and atom coordinates of nanosized RHO zeolite frozen while hydrated, and at 248 K without water molecules, are summarized in Figure 3d and Table S4 (Supporting Information), Figure 3e and Table S5 (Supporting Information), respectively. For nanosized RHO frozen at 248 K without water, the refined lattice parameter is *a* = 14.6655(8) Å (*I‐*43*m* space group), with a high ellipticity of 1.72 Å of the D8Rs, comparable to that of the activated nanosized RHO at 300 K (Table 2). Similarly, in the absence of water molecules at 248 K, the cationic positions remain consistent with those observed in the activated nanosized RHO at 300 K (Figure 3 and Tables S2 and S5, Supporting Information).

For hydrated nanosized RHO frozen at 248 K, the XRPD pattern (Figure 3d) shows along with the diffraction peaks corresponding hydrated RHO phase, peaks referable to ice phase, forming between the zeolite nanoparticles (Figure 3d; Figure S4, Supporting Information). The ice phase arises due to oversaturation of the nanosized RHO zeolite to ensure full hydration and ice peaks were excluded during the Rietveld refinement analysis. The presence of ice makes this refinement very challenging, however, the peak intensities and their ratios closely match with those observed at 300 K postfreezing (vide infra). These results provide meaningful insights into the structural arrangement of water molecules at subfreezing conditions. Unlike the two‐phase system observed for hydrated nanosized RHO at 300 K, the frozen hydrated RHO at 248 K exhibits a single expanded phase with a lattice parameter of *a* = 15.0924(4) Å (*I‐*43*m* space group, Table 2). The cations and water locations in the frozen hydrated RHO at 248 K remain similar to those in the hydrated RHO at 300 K. The Cs^+^ cations oscillate through the center of the D8Rs, coordinated with water molecules, while the Na^+^ cations is located at the center of the S6Rs and inside the *lta* cages, also coordinated with water molecules (Figure 4d and Table S4, Supporting Information).

Figure 3f and Table 2 and Table S6 (Supporting Information) present the XRPD pattern and Rietveld refinement of the hydrated nanosized RHO zeolite after being heated back to 300 K following freezing at 248 K, referred to as the metastable state at 300 K. The refined lattice parameter is *a* = 15.0936(4) Å (*I‐*43*m* space group), with a low D8R ellipticity of 1.07 Å, comparable to that of the frozen hydrated nanosized RHO at 248 K and to that hydrated at 300K. Notably, the hydrated‐frozen RHO zeolite upon reheating to 300 K exhibits predominantly a single expanded phase, in contrast to the initial hydrated nanosized RHO at 300 K, which displayed two distinct phases (Figure 3c vs Figure 3f). Consequently, this state is labeled the metastable state, as the expanded hydrated RHO unit cell remains stable even at 300 K (see Figure S5, Supporting Information). The cationic and water arrangements in the metastable state at 300 K closely resemble those in the hydrated RHO at 300 K. The Cs^+^ cations primarily oscillate within the D8Rs, interacting with water molecules, while the Na^+^ cations are distributed between the S6Rs and the *lta* cages, where they are also coordinated with water molecules (Figure 4f and Table S6, Supporting Information).

Finally, the XRPD patterns and Rietveld refinement of nanosized RHO zeolite reactivated at 500 and 300 K are summarized in Figure 3g and Table S7 (Supporting Information) and Figure 3h and Table S8 (Supporting Information), respectively. The refined lattice parameters are *a* = 14.7837(5) Å at 500 K and *a* = 14.7422(4) Å at 300 K, with corresponding D8R ellipticities of 1.69 and 1.70 Å, respectively (Table 2). The cationic positions in RHO reactivated at 500 K are identical to those in its initial activated state at 500 K (Tables S1 and S7, Supporting Information). Likewise, the cationic sites in RHO reactivated at 300 K match those of its initial activated state at 300 K (Tables S2 and S8, Supporting Information). This demonstrates that the fully expanded nanosized RHO zeolite in the metastable state at 300 K can reversibly contract upon heating to 500 K, reflecting the intrinsic flexibility of zeolite RHO.

The complementary experimental and computational approaches provide a comprehensive understanding of the structural response of hydrated and dehydrated nanosized RHO zeolite at subfreezing conditions. The DFT‐calculated unit cell parameters trend shows good agreement with the experimental one observed by in situ XRD, though with some quantitative differences. At 300 K, our simulations with 46 water molecules per unit cell predict a unit cell parameter of ≈15.14 Å (Table 1), while experimental measurements yield 15.09 and 14.84 (minor phase) Å (Table 2). This minor discrepancy (≈0.3%) may result from several factors: i) incomplete hydration of the experimental sample due to diffusion limitations within the nanocrystals, as suggested by Trzpit et al.,^[^ ^56^ ^]^ ii) the finite size effects inherent to DFT periodic boundary conditions, or iii) the limitations of GGA‐PBE functionals in precisely capturing hydrogen‐bonding interactions, as documented by Fischer.^[^ ^57^ ^]^ More significantly, both computational and experimental approaches capture the critical phase transition upon cooling the fully hydrated RHO framework (46 water molecules) to 248 K, albeit with differences in the transition dynamics (Tables 1 and 2). The DFT simulations predict a gradual transition to the expanded phase, while XRD patterns reveal a relatively abrupt transition from the two‐phase system to a single expanded phase. This difference is likely due to cooperative framework deformations that extend beyond a single unit cell, phenomena that our simulations cannot fully capture due to computational constraints. Nevertheless, the final structural state is consistently characterized by an expanded phase with *Im‐*3*m* symmetry, with both approaches showing ellipticity parameters (Δ) below 1.35 Å at 248 K (see Tables 1 and 2).

A particularly interesting observation from our simulations is the behavior of water molecules at subfreezing temperatures. Unlike bulk water that forms crystalline ice below 273 K, the confined water within the zeolite micropores maintains significant mobility even at 248 K, forming a “quasi‐liquid” state as reported in the literature for microporous materials.^[^ ^58^, ^59^ ^]^ This quasi‐liquid behavior explains the persistence of the expanded phase upon reheating to 300 K, as the confined water reorganizes into more stable hydrogen‐bonding networks that resist restructuring as we also observed using in situ FTIR spectroscopy (vide infra). The rearrangement of water molecules around cations provides the molecular‐level mechanism behind the experimentally observed metastability (Figures S2b and S3, Supporting Information). While our highest hydration model (46 water molecules) matches the theoretical maximum based on water adsorption measurement (Figure 1), the experimental sample may not have achieved complete saturation due to the relative humidity conditions (≈90%) used during hydration. This partial hydration scenario is better represented by our intermediate DFT models (37–40 water molecules), which show similar qualitative behavior but with slightly different unit cell parameters (14.90–15.03 Å at 300 K). Despite these quantitative differences, the consistency in the observed phase transition behavior across all hydrated models and the experimental data confirms the robustness of our findings regarding the low temperature‐induced stabilization of the expanded RHO phase.

### 3.3 In Situ FTIR Spectra of Nanosized RHO Zeolite at Subfreezing Conditions

**Figure 5:**
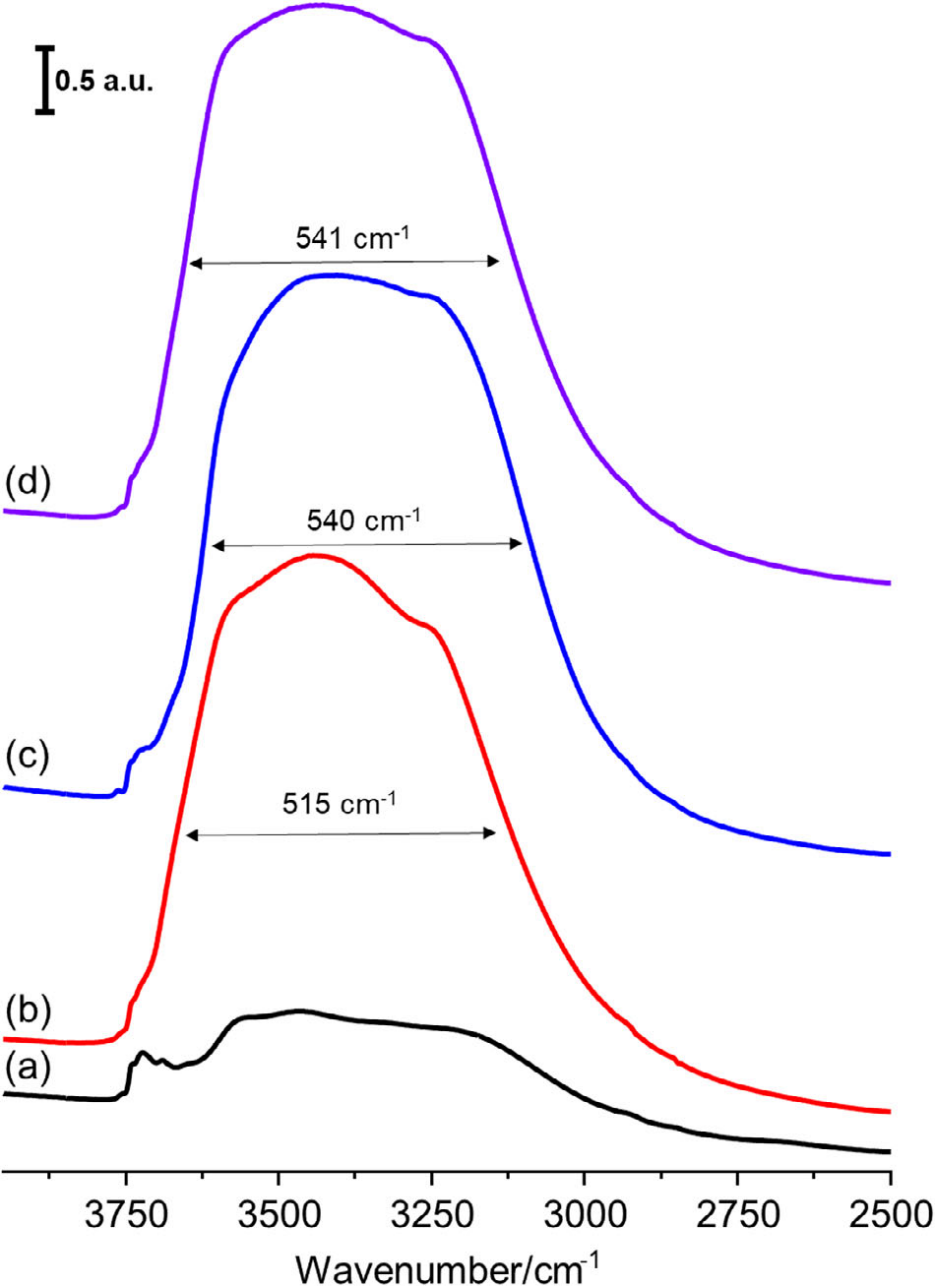
FTIR spectra of silanol region (2500–4000 cm^−1^) of nanosized RHO zeolite at 300 K a) activated, b) after adsorption of 8 ppm water, c) frozen with 8 ppm of water at 100 K, and consequently d) heated back to 300 K (meta state). The nanosized RHO zeolite was pretreated at 623 K under high vacuum (10^−6^ kPa) overnight prior recording the (a) FTIR spectrum.

To investigate the effect of water adsorption and freezing of the nanosized RHO zeolite, an in situ FTIR experiment was conducted, focusing on the silanol region of the FTIR spectra (**Figure** **5**). The zeolite was first activated at 623 K under vacuum overnight, and its FTIR spectrum was recorded (Figure 5a). The activated nanosized RHO exhibits a broad range of O─H stretching frequencies associated with T─OH groups, depending on hydrogen‐bonding strength. An enlarged FTIR spectrum (Figure S6, Supporting Information) highlights key peaks, although precise identification and quantification of silanol sites remain challenging due to the complex nature of the O─H stretching region.^[^ ^2^, ^60^ ^]^ Generally, lower O─H stretching frequencies correspond to shorter H‐bonds and stronger hydrogen bonding.^[^ ^2^, ^60^ ^]^ The activated zeolite exhibits: i) Isolated/weakly hydrogen‐bonded O─H (3758, 3740, 3724 cm^−1^), likely on the external surface; ii) Moderately hydrogen‐bonded O─H (3690, 3644, 3558 cm^−1^), and iii) Strongly hydrogen‐bonded O─H (broad peaks at 3490 and 3194 cm^−1^), likely inside the zeolite structure, attributed to silanol nests (T‐site defects) (Figure 5a; Figure S6, Supporting Information).^[^ ^2^ ^]^ After adsorbing 8 ppm of water at equilibrium, the FTIR spectrum at 300 K (Figure 5b) shows that the O─H stretching bands of water molecules overlap with those of the zeolite, leaving only a small portion of isolated silanol peaks (3758 and 3740 cm^−1^) visible. A broad water‐related peak appears between 3700 and 2800 cm^−1^ with a full width at half maximum (FWHM) of 515 cm^−1^. Subsequently, the hydrated zeolite was cooled to 100 K, and the FTIR spectrum was recorded (Figure 5c). Upon freezing, the O─H stretching band redistributes, shifting 35 cm^−1^ lower, and the FWHM increases to 540 cm^−1^. This redistribution suggests an increase in strongly hydrogen‐bonded O─H groups, hinting at a closer rearrangement of water molecules after freezing. When the frozen hydrated RHO was reheated to 300 K, the FTIR spectrum (Figure 5d) revealed that the broad O─H stretching peak shifted back 35 cm^−1^ to higher wavenumbers, similar to the hydrated state at 300 K (Figure 5b). However, the broader peak distribution (FWHM = 541 cm^−1^) and its shift toward lower frequencies remained consistent with the frozen state (Figure 5c). This suggests that the rearrangement of water molecules persists even after reheating, which aligns with the single expanded phase observed in the metastable state at 300 K (Figures 3f and 4f). Importantly, this persistence of rearranged water molecules within the zeolite microporosity helps to maintain the less distorted shape of the D8Rs, making the zeolite microporosity more accessible to guest molecules. This insight suggests that small quantities of retained water molecules could reduce the need for energy‐intensive activation methods, representing a green chemistry advancement that eliminates high‐temperature treatments for acceptable zeolite performance.

**Figure 6:**
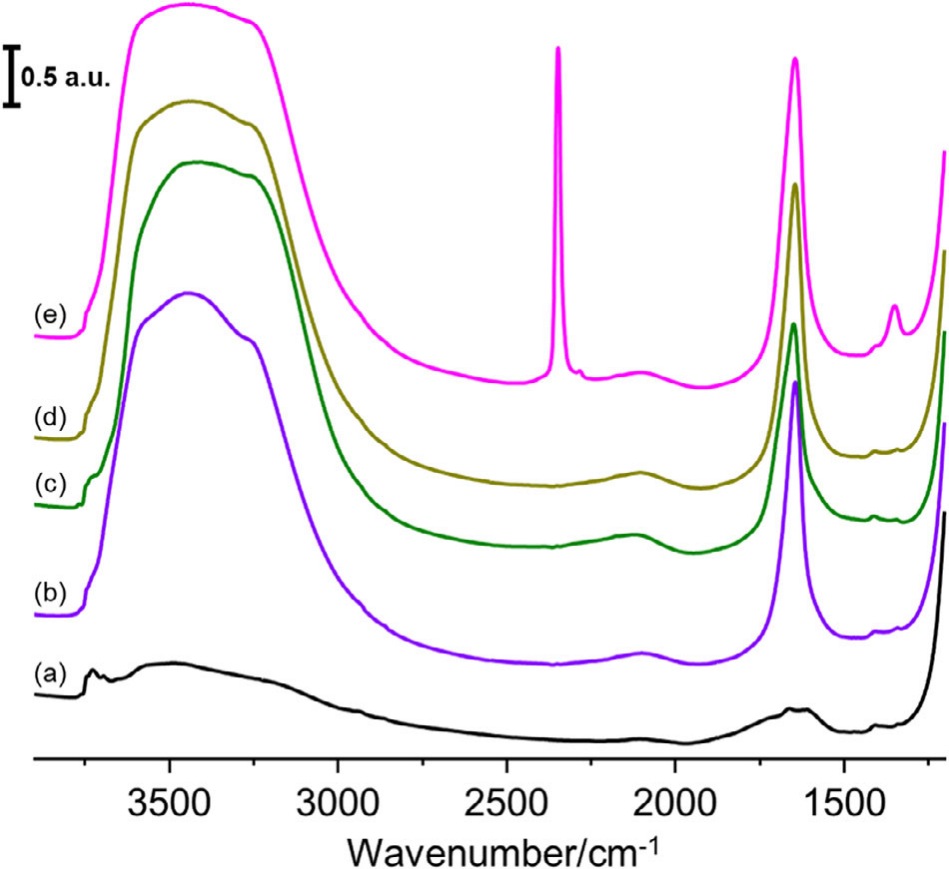
FTIR spectra of nanosized RHO zeolite a) activated, b) loaded with 8 ppm of water at 300 K, then c) cooled down to 100 K and d) bring back to 300 K (metastable state), and finally e) loaded with CO_2_ at 2.67 kPa and 300 K.

To assess whether the metastable state can be utilized in gas separation applications, specifically by enabling light activation methods instead of energy‐intensive heating treatments, 8 ppm of water was adsorbed onto nanosized RHO zeolite at equilibrium at 300 K, followed by cooling to 100 K and reheating to 300 K. Subsequently, 2.67 kPa of CO_2_ was introduced at 300 K, and the resulting FTIR spectra are presented in **Figure** **6**. The O─H stretching region (4000–2500 cm^−1^) has already been discussed in detail (vide supra). Upon water adsorption, a peak at 1643 cm^−1^ appears, corresponding to the bending mode of adsorbed water (Figure 6b).^[^ ^16^ ^]^ Upon CO_2_ adsorption, two peaks emerge at 2345 and 2279 cm^−1^, assigned to physisorbed ^12^CO_2_ and ^13^CO_2_, respectively (Figure 6e).^[^ ^3^ ^]^ Additionally, chemisorbed CO_2_ is identified by the presence of peaks at 1405 and 1349 cm^−1^ (Figure 6e).^[^ ^3^ ^]^ To quantify the CO_2_ adsorption, the ^13^CO_2_ peak (2279 cm^−1^) was integrated and corrected for the natural abundance of ^13^C (1.11%) to account for both ^12^CO_2_ and ^13^CO_2_.^[^ ^3^ ^]^ Similarly, the 1405 and 1349 cm^−1^ peaks were used to quantify chemisorbed CO_2_.^[^ ^3^ ^]^ Under 2.67 kPa of CO_2_, the estimated uptake was 0.44 mmol g^−1^ for physisorbed CO_2_ and 0.04 mmol g^−1^ for chemisorbed CO_2_, resulting in a total CO_2_ uptake of 0.48 mmol g^−1^. These values closely match those obtained from CO_2_ adsorption isotherms at 298 K (Figure 1c), where a CO_2_ uptake of 0.52 mmol g^−1^ was recorded at 2.67 kPa after high‐temperature activation (623 K overnight, 10^−1^ kPa vacuum). This agreement underscores the potential of the metastable state for gas separation applications, as it enables comparable CO_2_ uptake without requiring extensive thermal activation. These results are consistent with the recent work by Lee et al., where they demonstrated a rapid uptake of CO_2_ by Cs‐RHO zeolite loaded with H_2_O, linked to a cooperative effect of CO_2_/H_2_O on the movement of Cs^+^ cations away from the center of the D8Rs.^[^ ^61^ ^]^

Our findings demonstrate that the structural response of nanosized RHO zeolite to hydration and subfreezing cooling is highly reversible, yet distinct from previously observed high‐temperature transformations. Upon cooling to 248 K, the hydrated zeolite forms a single expanded phase (Figure 3d), which remains stable even after reheating to 300 K (Figure 3f), marking the formation of a metastable state. In situ FTIR analysis reveals that the water molecules within the zeolite micropores undergo reorganization at low temperatures, leading to persistent hydrogen‐bonding interactions upon re‐heating to 300 K (Figure 5). The metastable state exhibits a CO_2_ adsorption capacity comparable to that of conventionally activated RHO zeolite, demonstrating its feasibility as a green chemistry‐aligned low‐energy activation alternative (Figure 6). These findings establish new insight on hydration, freezing, and zeolite flexibility, providing new vistas for designing energy‐efficient gas separation processes.

## 4 Conclusion

While RHO‐type zeolite has been the subject of many experimental and theoretical investigations of its structural flexibility at ambient and elevated temperatures, the structural response of the framework at low (<273 K) temperatures has been significantly overlooked. In this study, we provide new insights into the structural behavior of nanosized RHO zeolite under subfreezing conditions with water molecules both absent and present in the microporosity. We demonstrate that water adsorption and subsequent freezing induce a metastable state at 300 K, characterized by a single expanded phase. Notably, the freezing of the water‐saturated zeolite induces subtle changes to the cation positions and occupancies, coincident with the rearrangement of the confined water molecules and their hydrogen bonding network, which persists upon reheating of the system to 300 K. This metastable state exhibits a CO_2_ equilibrium adsorption capacity comparable to the conventionally activated nanosized RHO zeolite, but without requiring high‐temperature treatments. This is likely linked to the less distorted D8R pore apertures, and cooperative CO_2_/H_2_O effect on the Cs^+^ cations. Our findings suggest that green chemistry‐inspired mild activation methods, such as moderate temperature treatments (<473 K) or medium vacuum levels (10^−1^ kPa), could be leveraged to enhance zeolite performance while embodying green chemistry principles of energy efficiency. The flexibility of nanosized RHO zeolite open new green chemistry possibilities for its application in sustainable gas separation and storage processes. From a green chemistry perspective, this work demonstrates multiple sustainable advantages that contribute to environmentally responsible zeolite processing. The green synthesis approach utilized for nanosized RHO zeolite synthesis eliminates organic structure‐directing agents, reducing synthesis complexity and environmental impact while achieving high crystalline yields. The nanosized nature of the crystals inherently provides green chemistry benefits through enhanced diffusion properties and reduced mass transfer limitations, improving gas separation efficiency compared to conventional micron‐sized zeolites. Most significantly, the subfreezing activation methodology represents a green chemistry innovation that replaces energy‐intensive high‐temperature treatments with mild cooling conditions, directly addressing green chemistry principles of energy efficiency and inherently safer processing. This approach eliminates the need for thermal treatments at elevated temperatures (623 K), reducing associated CO_2_ emissions and energy consumption in zeolite activation processes.

## Conflict of Interest

The authors declare no conflict of interest.

## Author Contributions

S.G. performed analysis, validation, visualization, conceptualization, writing of the original draft, and review and editing. G.C. conducted analysis, validation, visualization, and writing – review and editing. S.P.G. carried out analysis, validation, and review. E.C. performed synthesis, validation, and writing – review and editing. A.M. handled analysis and validation. D.P. conducted analysis, validation, and review and editing. F.D. performed analysis and validation. R.F. carried out analysis and validation. R.A. provided validation and resources and contributed to writing – review and editing. P.P. conducted analysis, validation, and editing. G.V. carried out analysis, validation, and provided resources and writing – review and editing. S.M. contributed to conceptualization, validation, project administration, resources, funding acquisition, and writing – review and editing.

## Supporting information



Supporting Information

## Data Availability

The data that support the findings of this study are available on request from the corresponding author. The data are not publicly available due to privacy or ethical restrictions.
