# Supplementary material for: Structural Flexibility of Hydrated RHO Nanosized Zeolite Synthesized via Green Synthesis Approach at Subfreezing Conditions
Source: Small Methods. 2025 Sep 4;9(11):e01376. doi: 10.1002/smtd.202501376 (PMC12641365; doi:10.1002/smtd.202501376)
Supplement: Supplementary file 1 — Supporting Information [file SMTD-9-e01376-s001.docx]

**Supporting Information**

**Structural flexibility of hydrated RHO nanosized zeolite synthesized via green synthesis approach at subfreezing conditions**

Sajjad Ghojavand^a*^, Giorgia Confalonieri^b^, Stoyan P. Gramatikov^c^, Edwin B. Clatworthy^a^, Aymeric Magisson^a^, Diógenes Honorato Piva^a^, Francesco Dalena^a^, Riccardo Fantini^d^, Rossella Arletti^d^, Petko St. Petkov^c^, Georgi N. Vayssilov^c^, Svetlana Mintova^a,c*^

^a^Université de Caen Normandie, ENSICAEN, CNRS, LCS, Laboratoire Catalyse et Spectrochimie, 14000 Caen, France

^b^Department of Earth Sciences, Sapienza Università di Roma, 00185 Rome, Italy

^c^Faculty of Chemistry and Pharmacy, Sofia University “St. Kliment Ohridski”, 1126 Sofia, Bulgaria ^d^Chemical and Geological Sciences Department, University of Modena and Reggio Emilia, Via G. Campi 103, Modena 41125, Italy

^*^Corresponding authors. E-mail addresses: [sajjad.ghojavand@ensicaen.fr](mailto:sajjad.ghojavand@ensicaen.fr), +33231451330

[svetlana.mintova@ensicaen.fr](mailto:svetlana.mintova@ensicaen.fr), +33231452737


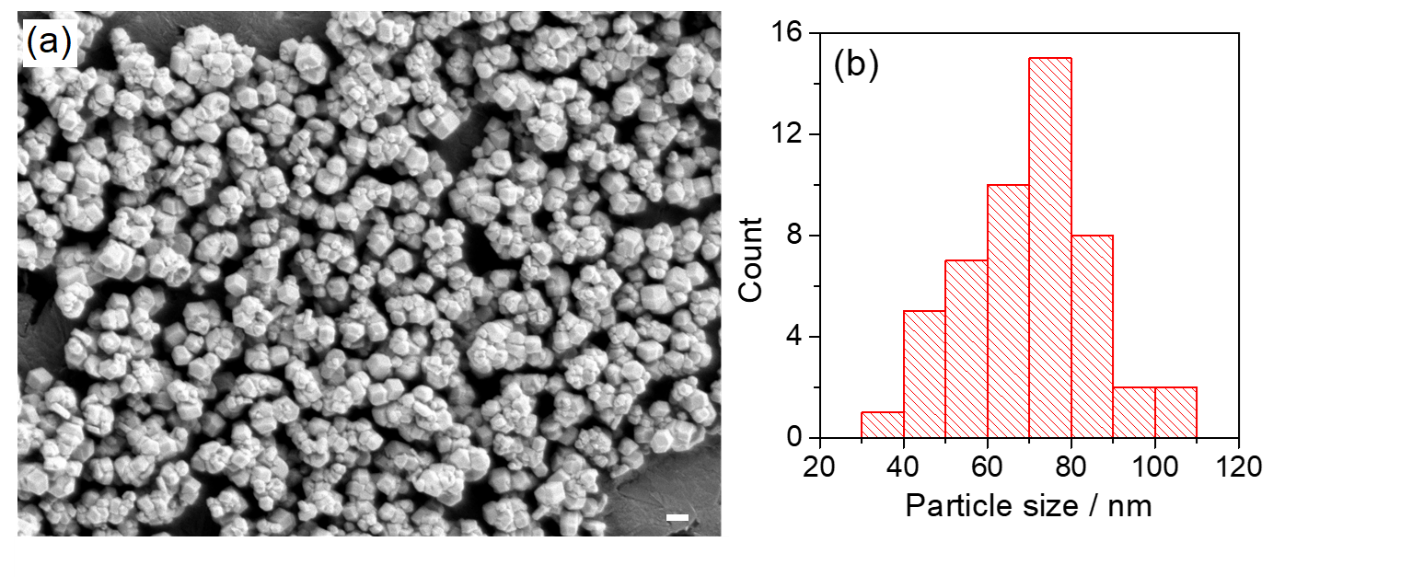


Fig. S1. SEM image and the corresponding particle size distribution of nanosized RHO zeolite.


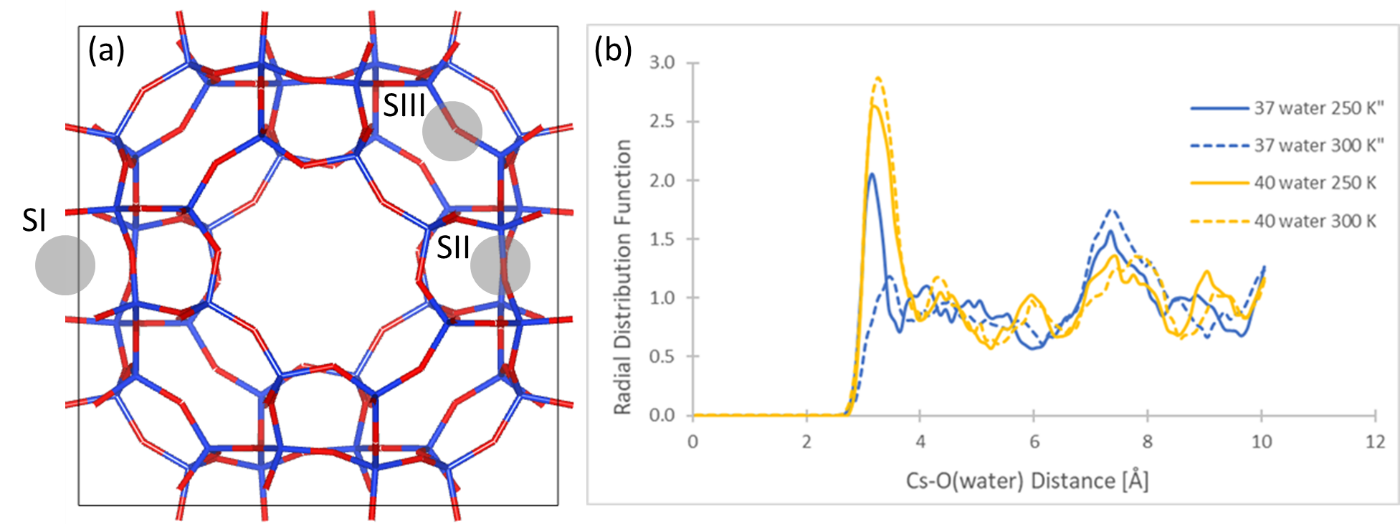


Fig. S2. (a) A schematic initial RHO structure representation highlighting the cationic sites, SI (in the middle of D8Rs), SII in S8Rs, and SIII in S6Rs. (b) Plot of the radial distribution functions of the distance between the Cs^+^ extra-framework cations and an oxygen atom of H_2_O for RHO structural models used in simulations.

| 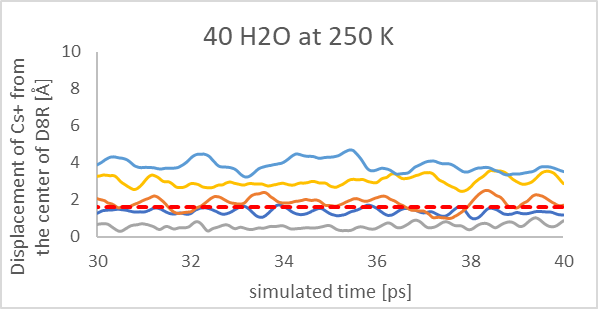 | 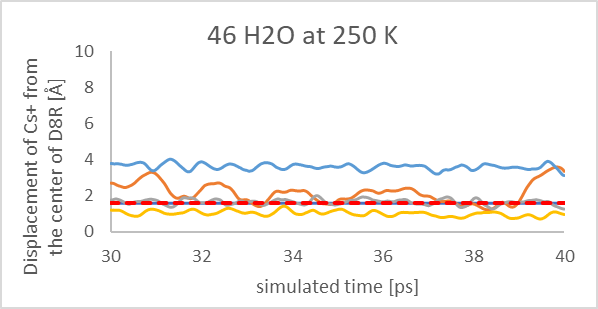 |
| --- | --- |
| 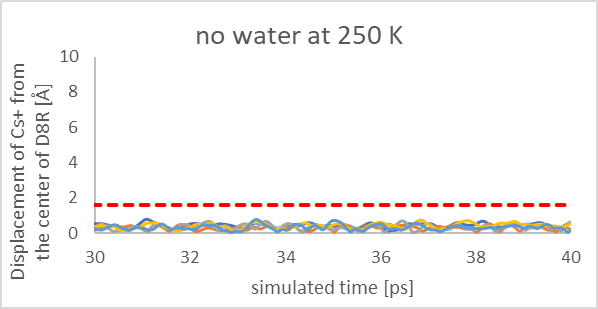  Fig. S3. Plot of the displacement of Cs^+^ from the center of the *D8R*s from the last 10 ps of the trajectories, shown in Å (different colors represent five different Cs^+^ cations present in the nanosized RHO structure). The red dashed line indicates the half distance between the two 8MR of the d8r –1.6 Å. | |

Table S1. Details of XRPD data and Rietveld refinement (including the framework/extra-framework distances in Å) of nanosized RHO activated at 500 K.

| **Atom** | **x** | **y** | **z** | **Occupancy** | **B (Å^2^)** |
| --- | --- | --- | --- | --- | --- |
| Si1 | 0.88043(8) | 0.72935(9) | 0.57805(8) | 0.654 | 0.74(2) |
| Al1 | 0.88043(8) | 0.72935(9) | 0.57805(8) | 0.346 | 0.74(2) |
| O1 | 0.88837(18) | 0.70981(19) | 0.46817(15) | 1 | 0.92(3) |
| O2 | 0.8794(3) | 0.63256(17) | 0.63256(17) | 1 | 0.92(3) |
| O3 | 0.7854(1) | 0.7854(1) | 0.5994(3) | 1 | 0.92(3) |
| Cs1 | 0.1993(14) | 0.5 | 0.5 | 0.033(1) | 4.82(3) |
| Cs2 | 0 | 0.5 | 0.5 | 0.768(2) | 4.82(3) |
| Na1 | 0.3086(11) | 0.5573(8) | 0.5573(8) | 0.150(2) | 2.9(1) |
| Na2 | 0.19923(18) | 0.80077(18) | 0.80077(18) | 1.00(1) | 2.9(1) |
| Framework/extra-framework distances/Å | | | | | |
| Cs1 O2 x1 | 2.998(9) | Na2 O3 x3 | 2.304(4) | Na2 O2 x3 | 2.984(4) |

Table S2. Details of XRPD data and Rietveld refinement (including the framework/extra-framework distances in Å) of nanosized RHO activated at 300 K.

| **Atom** | **x** | **y** | **z** | **Occupancy** | **B (Å^2^)** |
| --- | --- | --- | --- | --- | --- |
| Si1 | 0.88098(7) | 0.72976(8) | 0.57818(8) | 0.654 | 0.79(2) |
| Al1 | 0.88098(7) | 0.72976(8) | 0.57818(8) | 0.346 | 0.79(2) |
| O1 | 0.88903(17) | 0.7087(18) | 0.46856(15) | 1 | 1.00(3) |
| O2 | 0.8802(3) | 0.63392(17) | 0.63392(17) | 1 | 1.00(3) |
| O3 | 0.78581(16) | 0.78581(16) | 0.5987(3) | 1 | 1.00(3) |
| Cs1 | 0.194(2) | 0.5 | 0.5 | 0.0186(7) | 4.18(3) |
| Cs2 | 0 | 0.5 | 0.5 | 0.796(2) | 4.18(3) |
| Na1 | 0.3118(11) | 0.5565(8) | 0.5565(8) | 0.150(2) | 3.0(9) |
| Na2 | 0.19819(18) | 0.80181(18) | 0.80181(18) | 1.00(1) | 3.0(9) |
| Framework/extra-framework distances/Å | | | | | |
| Cs1 O2 x1 | 2.997(4) | Na2 O3 x3 | 2.341(4) | Na2 O2 x3 | 2.997(4) |

Table S3. Details of XRPD data and Rietveld refinement (including the framework/extra-framework and extra-framework/extra-framework distances in Å) of hydrated nanosized RHO nanozeolite at 300 K.

| **Atom** | **x** | **y** | **z** | **Occupancy** | **B (Å^2^)** |
| --- | --- | --- | --- | --- | --- |
| Si1 | 0.88452(10) | 0.73676(10) | 0.59163(9) | 0.654 | 1.12(3) |
| Al1 | 0.88452(10) | 0.73676(10) | 0.59163(9) | 0.346 | 1.12(3) |
| O1 | 0.8801(3) | 0.7242(2) | 0.4829(3) | 1 | 2.61(5) |
| O2 | 0.8683(4) | 0.6400(3) | 0.6400(3) | 1 | 2.61(5) |
| O3 | 0.8069(3) | 0.8069(3) | 0.6235(4) | 1 | 2.61(5) |
| Cs1 | 0.1481(8) | 0.5 | 0.5 | 0.157(3) | 2.61(8) |
| Cs2 | 0.0982(6) | 0.5 | 0.5 | 0.185(2) | 2.61(8) |
| Cs3 | 0.0262(8) | 0.5 | 0.5 | 0.0750(1) | 2.61(8) |
| Na1 | 0.2957(7) | 0.5672(6) | 0.5672(6) | 0.407(3) | 9.3(3) |
| Na2 | 0.251(2) | 0.251(2) | 0.251(2) | 0.229(8) | 9.3(3) |
| W1 | 0.3276(5) | 0.3276(5) | 0.3276(5) | 0.738(18) | 4.4(1) |
| W2 | 0.172(1) | 0.5 | 0.5 | 0.58(1) | 4.4(1) |
| W3 | 0.7990(5) | 0.7990(5) | 0.0054(9) | 0.582(8) | 4.4(1) |
| W4 | 0 | 0 | 0 | 1.00(2) | 4.4(1) |
| W5 | 0.580(1) | 0.6467(8) | 0.6467(8) | 0.333(7) | 4.4(1) |
| Framework/extra-framework distances/Å | | | | | |
| Cs1 O2 x2 | 2.998(6) | Cs2 O2 x2 | 3.030(6) | Na2 O3 x3 | 2.264(9) |
| Na2 O2 x3 | 2.960(2) | W1 O3 x3 | 3.112(11) | W2 O2 x2 | 3.049(7) |
| W3 O1 x2 | 2.747(9) | W3 O3 | 2.880(13) |  |  |
| Extra-framework/extra-framework distances/Å | | | | | |
| Cs3 W2 | 2.990(2) | Na1 W1 | 2.297(15) | Na1 W3 x2 | 2.298(10) |
| Na1 W2 | 2.350(2) | Na1 W5 x2 | 2.670(17) | W1 W3 x3 | 2.752(14) |
| W3 W5 x2 | 2.925(18) |  |  |  |  |

Table S4. Details of XRPD data and Rietveld refinement (including the framework/extra-framework and extra-framework/extra-framework distances in Å) of hydrated nanosized RHO at 248 K (frozen). Results presented are considered tentative due to the presence of ice, as explained in the main text.

| **Atom** | **x** | **y** | **z** | **Occupancy** | **B (Å^2^)** |
| --- | --- | --- | --- | --- | --- |
| Si1 | 0.8846(1) | 0.7374(1) | 0.5918(1) | 0.654 | 0.89(3) |
| Al1 | 0.8846(1) | 0.7374(1) | 0.5918(1) | 0.346 | 0.89(3) |
| O1 | 0.8800(3) | 0.7249(2) | 0.4835(3) | 1 | 1.94(6) |
| O2 | 0.8670(4) | 0.6406(3) | 0.6406(3) | 1 | 1.94(6) |
| O3 | 0.8086(3) | 0.8086(3) | 0.62345(4) | 1 | 1.94(6) |
| Cs1 | 0.1393(7) | 0.5 | 0.5 | 0.157(2) | 2.50(8) |
| Cs2 | 0.0936(6) | 0.5 | 0.5 | 0.191(2) | 2.50(8) |
| Cs3 | 0.0220(1) | 0.5 | 0.5 | 0.069(8) | 2.50(8) |
| Na1 | 0.2948(6) | 0.5575(5) | 0.5575(5) | 0.390(2) | 9.1(9) |
| Na2 | 0.25145(11) | 0.25145(11) | 0.25145(11) | 0.279(6) | 9.1(9) |
| W1 | 0.3395(6) | 0.3395(6) | 0.3395(6) | 0.70(1) | 4.0(2) |
| W2 | 0.1702(9) | 0.5 | 0.5 | 0.58(1) | 4.0(2) |
| W3 | 0.8025(4) | 0.8025(4) | 0.0019(9) | 0.66(1) | 4.0(2) |
| W4 | 0 | 0 | 0 | 1.00(1) | 4.0(2) |
| W5 | 0.5873(9) | 0.6488(7) | 0.6488(7) | 0.333(5) | 4.0(2) |
| Framework/extra-framework distances/Å | | | | | |
| Cs1 O2 x2 | 3.002(6) | Cs2 O2 x2 | 3.059(6) | Na2 O3 x3 | 2.285(3) |
| Na2 O2 x2 | 2.939(6) | W2 O2 x2 | 3.053(7) | W3 O1 x2 | 2.800(9) |
| W3 O3 | 2.998(12) |  |  |  |  |
| Extra-framework/extra-framework distances/Å | | | | | |
| Na1 W1 | 2.299(13) | Na1 W3 x2 | 2.247(17) | Na1 W5 x2 | 2.720(17) |
| Na2 W1 | 2.301(16) | W1 W3 x3 | 2.575(14) | W3 W5 x2 | 2.866(17) |

Table S5. Details of XRPD data and Rietveld refinement (including the framework/extra-framework in Å) of nanosized RHO at 248 K without water.

| **Atom** | **x** | **y** | **z** | **Occupancy** | **B (Å^2^)** |
| --- | --- | --- | --- | --- | --- |
| Si1 | 0.88025(11) | 0.72906(12) | 0.57778(11) | 0.654 | 0.74(3) |
| Al1 | 0.88025(11) | 0.72906(12) | 0.57778(11) | 0.346 | 0.74(3) |
| O1 | 0.8885(2) | 0.7092(2) | 0.4677(2) | 1 | 0.93(5) |
| O2 | 0.8799(3) | 0.6325(2) | 0.6325(2) | 1 | 0.93(5) |
| O3 | 0.7845(2) | 0.7845(2) | 0.5989(3) | 1 | 0.93(5) |
| Cs1 | 0.202(7) | 0.5 | 0.5 | 0.018 | 3.82(4) |
| Cs2 | 0 | 0.5 | 0.5 | 0.794 | 3.82(4) |
| Na1 | 0.3214(13) | 0.5490(10) | 0.5490(10) | 0.15 | 2.5(1) |
| Na2 | 0.1947(2) | 0.8053(2) | 0.8053(2) | 1 | 2.5(1) |
| Framework/extra-framework distances/Å | | | | | |
| Cs1 O2 x2 | 3.000(4) | Na2 O3 x3 | 2.333(5) | Na2 O2 x3 | 3.006(5) |


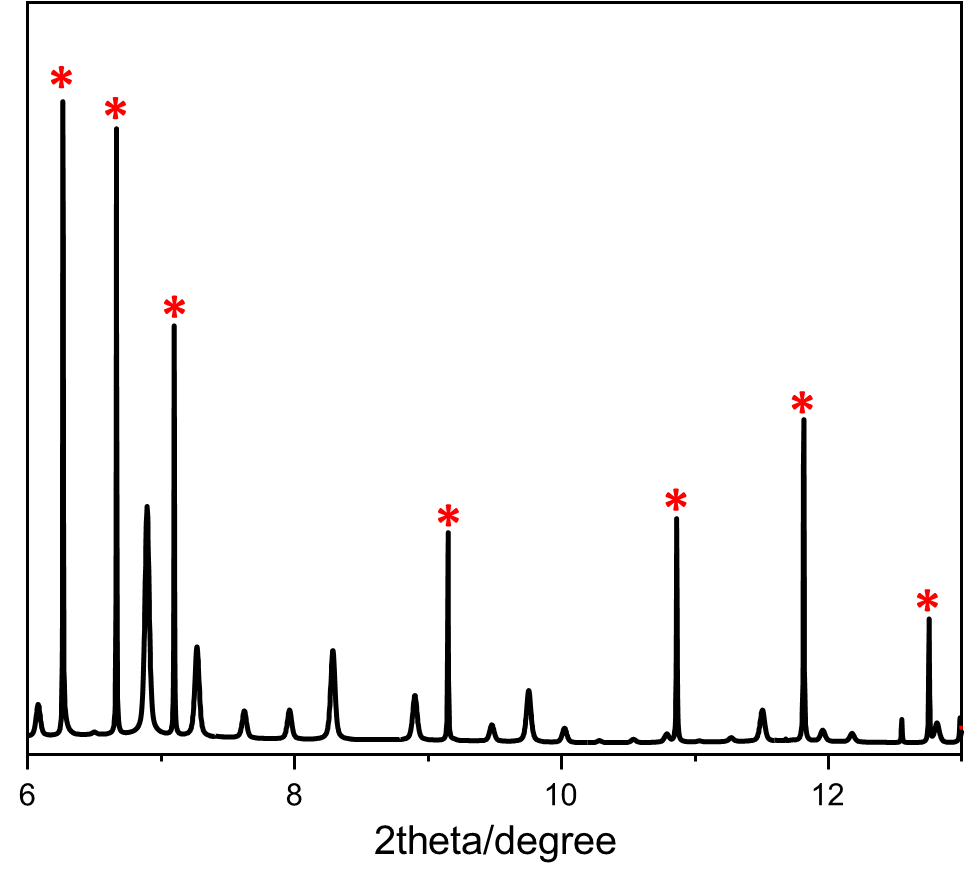


Fig. S4. XRPD pattern of hydrated nanosized RHO zeolite at 248 K. Red asterisks highlight the diffraction peaks due to ice formation between nanosized RHO particles.

Table S6. Details of XRPD data and Rietveld refinement (including the framework/extra-framework and extra-framework/extra-framework distances in Å) of hydrated nanosized RHO at 300 K after freezing water inside the pores at 248 K (metastable).

| **Atom** | **x** | **y** | **z** | **Occupancy** | **B (Å^2^)** |
| --- | --- | --- | --- | --- | --- |
| Si1 | 0.88482(8) | 0.73769(8) | 0.59207(7) | 0.654 | 0.94(3) |
| Al1 | 0.88482(8) | 0.73769(8) | 0.59207(7) | 0.346 | 0.94(3) |
| O1 | 0.8800(2) | 0.72486(18) | 0.48371(19) | 1 | 2.10(4) |
| O2 | 0.8669(3) | 0.6411(2) | 0.6411(2) | 1 | 2.10(4) |
| O3 | 0.8090(2) | 0.8090(2) | 0.6234(3) | 1 | 2.10(4) |
| Cs1 | 0.1394(6) | 0.5 | 0.5 | 0.190(3) | 3.52(7) |
| Cs2 | 0.0886(7) | 0.5 | 0.5 | 0.163(2) | 3.52(7) |
| Cs3 | 0.0206(12) | 0.5 | 0.5 | 0.064(2) | 3.52(7) |
| Na1 | 0.2950(6) | 0.5574(5) | 0.5574(5) | 0.385(2) | 9.0(3) |
| Na2 | 0.2517(14) | 0.2517(14) | 0.2517(14) | 0.294(7) | 9.0(3) |
| W1 | 0.3397(5) | 0.3397(5) | 0.3397(5) | 0.706(14) | 4.0(1) |
| W2 | 0.1739(10) | 0.5 | 0.5 | 0.583(11) | 4.0(1) |
| W3 | 0.8033(3) | 0.8033(3) | 0.0034(6) | 0.630(6) | 4.0(1) |
| W4 | 0 | 0 | 0 | 1.00(2) | 4.0(1) |
| W5 | 0.58333(10) | 0.6469(7) | 0.6469(7) | 0.333(6) | 4.0(1) |
| Framework/extra-framework distances/Å | | | | | |
| Cs1 O2 x2 | 3.013(4) | Cs2 O2 x2 | 3.085(5) | Na2 O3 x3 | 2.289(6) |
| Na2 O2 x2 | 2.933(13) | W2 O2 x2 | 3.073(5) | W3 O1 x2 | 2.815(7) |
| W3 O3 | 3.004(9) |  |  |  |  |
| Extra-framework/extra-framework distances/Å | | | | | |
| Cs3 W2 | 2.940(2) | Na1 W1 | 2.298(12) | Na1 W3 x2 | 2.298(7) |
| Na1 W5 x2 | 2.665(13) | Na2 W1 | 2.300(4) | W1 W3 x3 | 2.591(10) |
| W3 W5 x2 | 2.860(13) |  |  |  |  |


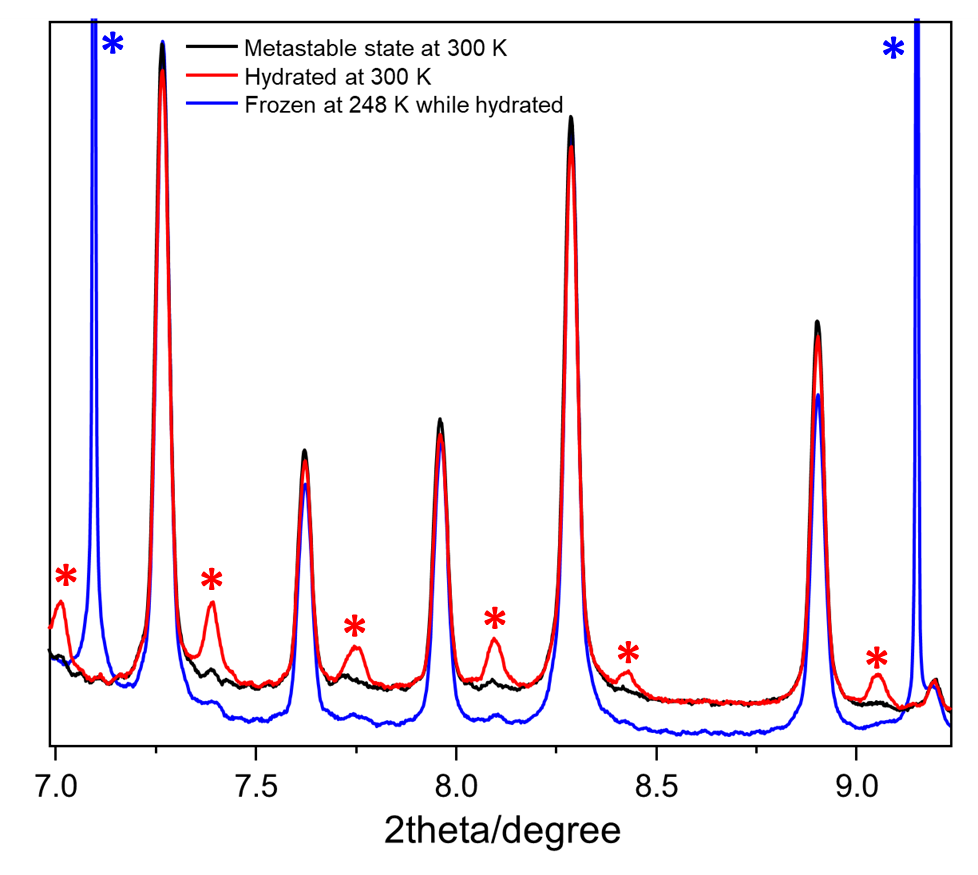


Fig. S5. XRPD pattern of hydrated nanosized RHO zeolite at 300 K before and after freezing and frozen at 248 K while hydrated (the blue asterisks represent the interparticle ice XRD pattern and the red asterisks highlights the secondary hydrated phase in nanosized RHO zeolite).

Table S7. Details of XRPD data and Rietveld refinement (including the framework/extra-framework in Å) of nanosized RHO reactivated at 500 K.

| **Atom** | **x** | **y** | **z** | **Occupancy** | **B (Å^2^)** |
| --- | --- | --- | --- | --- | --- |
| Si1 | 0.88067(8) | 0.72968(9) | 0.57832(9) | 0.654 | 1.20(2) |
| Al1 | 0.88067(8) | 0.72968(9) | 0.57832(9) | 0.346 | 1.20(2) |
| O1 | 0.88834(19) | 0.71011(19) | 0.46865(16) | 1 | 1.47(4) |
| O2 | 0.8792(3) | 0.6329(18) | 0.6329(18) | 1 | 1.47(4) |
| O3 | 0.7862(2) | 0.7862(2) | 0.5998(3) | 1 | 1.47(4) |
| Cs1 | 0.197(1) | 0.5 | 0.5 | 0.0401(8) | 6.24(4) |
| Cs2 | 0 | 0.5 | 0.5 | 0.753(2) | 6.24(4) |
| Na1 | 0.3253(1) | 0.6307(7) | 0.6307(7) | 0.150(8) | 3.7(1) |
| Na2 | 0.2047(2) | 0.7953(2) | 0.7953(2) | 1.00(5) | 3.7(1) |
| Framework/extra-framework distances/Å | | | | | |
| Cs1 O2 x2 | 2.998(9) | Na2 O3 x2 | 2.306(4) | Na2 O2 x3 | 2.984(4) |

Table S8. Details of XRPD data and Rietveld refinement (including the framework/extra-framework in Å) of nanosized RHO reactivated at 300 K.

| **Atom** | **x** | **y** | **z** | **Occupancy** | **B (Å^2^)** |
| --- | --- | --- | --- | --- | --- |
| Si1 | 0.88064(8) | 0.72968(8) | 0.57836(9) | 0.654 | 1.14(2) |
| Al1 | 0.88064(8) | 0.72968(8) | 0.57836(9) | 0.346 | 1.14(2) |
| O1 | 0.88827(18) | 0.71020(19) | 0.46866(16) | 1 | 1.50(3) |
| O2 | 0.8791(3) | 0.63284(17) | 0.63284(17) | 1 | 1.50(3) |
| O3 | 0.78623(16) | 0.78623(16) | 0.5999(3) | 1 | 1.50(3) |
| Cs1 | 0.1972(13) | 0.5 | 0.5 | 0.040(1) | 6.52(4) |
| Cs2 | 0 | 0.5 | 0.5 | 0.753(2) | 6.52(4) |
| Na1 | 0.3281(9) | 0.6278(7) | 0.6278(7) | 0.150(2) | 3.4(1) |
| Na2 | 0.20429(19) | 0.79571(19) | 0.79571(19) | 1.00(5) | 3.4(1) |
| Framework/extra-framework distances/Å | | | | | |
| Cs1 O2 x2 | 2.997(9) | Na2 O3 x3 | 2.322(4) | Na2 O2 x3 | 2.998(4) |

Table S9. Details of structural refinement parameters.

|  | **RHO activated at 500 K** | **RHO activated at 300 K** | **Hydrated RHO at 300 K*** | **Hydrated RHO at 248 K (frozen)*** | **RHO at 248 K without water** | **RHO at 300 K after freezing** | **RHO reactivated at 500 K** | **RHO reactivated at 300 K** |
| --- | --- | --- | --- | --- | --- | --- | --- | --- |
| **Rwp(%)** | 5.20 | 5.40 | 3.84 | 4.87 | 6.17 | 3.48 | 5.15 | 4.76 |
| **R_exp_(%)** | 0.64 | 0.64 | 0.55 | 0.56 | 0.72 | 0.56 | 0.62 | 1.22 |
| **Rp(%)** | 4.87 | 5.02 | 3.10 | 3.90 | 5.70 | 2.65 | 4.80 | 4.67 |
| **No. of**  **variables** | 82 | 82 | 92 | 90 | 82 | 90 | 82 | 82 |
| **No. of**  **reflections** | 480 | 478 | 509 | 509 | 473 | 509 | 480 | 478 |

*Excluding Le Bail no. of variables


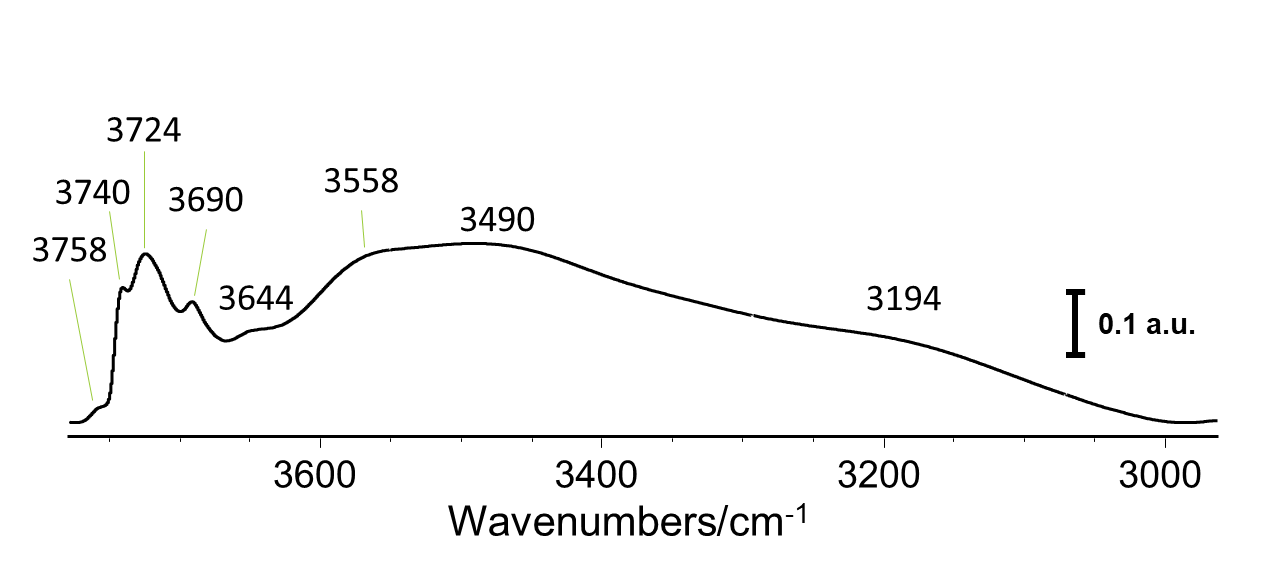


Fig. S6. FTIR spectrum of the silanol region (3000–4000 cm^-1^) of the activated nanosized RHO zeolite, recorded at 300 K. The nanosized RHO zeolite was pretreated at 623 K under high vacuum (10^-6^ kPa) overnight prior to the measurement.
